# Immunogenic Cell Death Activates the Tumor Immune Microenvironment to Boost the Immunotherapy Efficiency

**DOI:** 10.1002/advs.202201734

**Published:** 2022-06-02

**Authors:** Zhilin Li, Xiaoqin Lai, Shiqin Fu, Long Ren, Hao Cai, Hu Zhang, Zhongwei Gu, Xuelei Ma, Kui Luo

**Affiliations:** ^1^ Department of Biotherapy Huaxi MR Research Center (HMRRC) Day Surgery Center Department of Radiology Cancer Center Research Core Facilities of West China Hospital National Clinical Research Center for Geriatrics Frontiers Science Center for Disease‐Related Molecular Network State Key Laboratory of Biotherapy West China Hospital Sichuan University Chengdu 610041 China; ^2^ Amgen Bioprocessing Centre Keck Graduate Institute Claremont CA 91711 USA; ^3^ Functional and Molecular Imaging Key Laboratory of Sichuan Province and Research Unit of Psychoradiology Chinese Academy of Medical Sciences Chengdu 610041 China

**Keywords:** antitumor, drug delivery system, immunogenic cell death, immunotherapy, nanomedicines, synergic therapy

## Abstract

Tumor immunotherapy is only effective in a fraction of patients due to a low response rate and severe side effects, and these challenges of immunotherapy in clinics can be addressed through induction of immunogenic cell death (ICD). ICD is elicited from many antitumor therapies to release danger associated molecular patterns (DAMPs) and tumor‐associated antigens to facilitate maturation of dendritic cells (DCs) and infiltration of cytotoxic T lymphocytes (CTLs). The process can reverse the tumor immunosuppressive microenvironment to improve the sensitivity of immunotherapy. Nanostructure‐based drug delivery systems (NDDSs) are explored to induce ICD by incorporating therapeutic molecules for chemotherapy, photosensitizers (PSs) for photodynamic therapy (PDT), photothermal conversion agents for photothermal therapy (PTT), and radiosensitizers for radiotherapy (RT). These NDDSs can release loaded agents at a right dose in the right place at the right time, resulting in greater effectiveness and lower toxicity. Immunotherapeutic agents can also be combined with these NDDSs to achieve the synergic antitumor effect in a multi‐modality therapeutic approach. In this review, NDDSs are harnessed to load multiple agents to induce ICD by chemotherapy, PDT, PTT, and RT in combination of immunotherapy to promote the therapeutic effect and reduce side effects associated with cancer treatment.

## Graphical abstract

This review summarizes the nanostructure‐based drug delivery systems‐based immunogenic cell death (ICD) induction approaches combined with immunotherapy. Engineered and functionalized NDDSs to enhance their delivery efficiency and activate the tumor immune microenvironment for improving immunotherapeutic efficiency are illustrated.

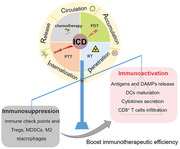

## 1 Introduction

Cancer immunotherapy through the use of immune checkpoint inhibitors (ICIs), lymphocyte activating cytokines, chimeric antigen receptor (CAR)‐T cells and other cells, cancer vaccines, oncolytic viruses, and bispecific antibodies has transformed the situation of cancer therapy.^[^ ^1^ ^]^ Immunotherapy has been a milestone of cancer treatment, which has been acknowledged with the Nobel Prize award for Physiology or Medicine in 2018 to James Alison and Tasuku Honjo who discovered immune checkpoints.^[^ ^2^ ^]^ Currently, ICIs have been approved including the antibody against cytotoxic T lymphocyte‐associated antigen‐4 (CTLA‐4), ipilimumab, in 2011, and blocking antibodies for programmed cell death protein1 (anti‐PD‐1) and its ligand 1 (anti‐PD‐L1), pembrolizumab and nivolumab, in 2014.^[^ ^3^ ^]^ Despite rapid development and great potential of cancer immunotherapy, only 10–30% of patients have gained the therapeutic benefit from ICIs due to unsatisfactory responses according to clinical reports.^[^ ^4^ ^]^ Patients also experience severe immune‐related adverse side effects, such as cytokines storm, pneumonitis, fetal myocarditis, and neurotoxic effects, which could be fatal due to off‐target distribution of ICIs.^[^ ^5^ ^]^ Such a low response to ICIs could be ascribed to “cold tumors” in the majority of patients, which are absent of tumor infiltrating T cells^[^ ^6^ ^]^ and have an immunosuppressive tumor microenvironment.^[^ ^7^ ^]^ Hence, great interests are drawn on developing synergistic therapeutic approaches to activate the tumor immune microenvironment to promote the immunotherapeutic efficacy.

One promising approach of activating the tumor immune microenvironment is induction of immunogenic cell death (ICD) in tumor cells. ICD can release antigens and danger associated molecular patterns (DAMPs) including calreticulin (CRT) on the tumor cell surface, as well as other factors such as high mobility group box 1 (HMGB1), adenosine‐5′‐triphosphate (ATP) and heat shock proteins (HSPs).^[^ ^8^ ^]^ CRT, a soluble protein on the endoplasmic reticulum (ER), could be translocated to the tumor cell surface under the ER stress.^[^ ^9^ ^]^ This translocation process is triggered by phosphorylating the eukaryotic translation initiation factor (eIF2*α*) via PKP‐like ER kinase (PERK) and activating proapoptotic proteins of BAX and BAK via caspase 8‐mediated B cell receptor associated protein 31 (BCAP31).^[^ ^10^ ^]^ CRT binding with the CD91 receptor acts as an “eat‐me” signal to facilitate antigen presenting cells (APCs) recognition of tumor associated antigens released by tumor cells;^[^ ^11^ ^]^ dying cells activate autophagy to secrete ATP that binds to P_2_X_7_ receptors on phagocytes,^[^ ^12^ ^]^ and this ATP binding process serves as a “find‐me” signal to recruit phagocytes like dendritic cells (DCs), macrophages and monocytes;^[^ ^13^ ^]^ HMGB1 interacts with toll‐like receptor 4 (TLR‐4) to facilitate DCs maturation and release proinflammatory cytokines to elicit potent immunostimulatory effects;^[^ ^14^ ^]^ and HSPs including HSP70 can attract phagocytes and activate natural killer (NK) cells.^[^ ^15^ ^]^ In general, the released tumor‐associated antigens are engulfed by APCs (such as DCs) and then presented to T cells, meanwhile those DAMPs facilitate maturation of DCs, activation of cytotoxic T lymphocytes (CTLs) and secretion of multiple cytokines (interferon‐*γ* (IFN‐*γ*), tumor necrosis factor‐*α* (TNF‐*α*), interleukin‐6 (IL‐6), interleukin‐1*β* (IL‐1*β*)) associated with innate and adaptive immunities to induce effective immune responses.^[^ ^16^ ^]^ The repertoire of antitumor T cells and cytokines transforms the tumor microenvironment and changes “cold” immunosuppressive tumors to “hot” immunoresponsive lesions, thus, increasing responses of the immunotherapy.^[^ ^17^ ^]^

It has been demonstrated that ICD within the tumor microenvironment can be induced from chemotherapy, photodynamic therapy (PDT), photothermal therapy (PTT), radiotherapy (RT).^[^ ^18^ ^]^ Although combinational therapy of these ICD‐inducing therapy and immunotherapy is a promising approach to regressing tumors and decreasing the recurrence rate, a conventional administration strategy could reduce its therapeutic effect. For instance, therapeutic agents may lose their therapeutic function under physiological conditions, have an insufficient circulation time, poor accumulation in tumor tissues, inadequate penetration into intratumoral tissues, and experience passive diffusion to cause damage and induce toxicity to normal cells.^[^ ^19^ ^]^ Therefore, the balance between their efficiency and toxicity should be elaborately maintained. Nanostructure‐based drug delivery systems (NDDSs) have demonstrated their great prospects in addressing the above challenges.^[^ ^20^ ^]^ They have specific features: 1) NDDSs can protect therapeutic agents from the physiological condition until they are delivered into targeted cells, which is especially important for macromolecular drugs such as antibodies and cytokines; 2) NDDSs can improve the solubility of hydrophobic therapeutic agents such as photosensitizers (PSs);^[^ ^21^ ^]^ 3) NDDSs can be engineered to be responsive in the tumor microenvironment, such as a low pH or reductive enzymes, and specific to target tumor cells by modification with unique antibody or peptide,^[^ ^22^ ^]^ which could reduce the toxicities of therapeutic agents to normal cells;^[^ ^23^ ^]^ 4) NDDSs can be designed to simultaneously load multiple therapeutic agents including small molecules, polypeptides or nucleic acids into the same carrier to achieve an “all in one” combinational therapy. Thus, NDDSs could be explored for ICD‐based synergic immunotherapies.^[^ ^24^ ^]^

Herein, the NDDSs‐based ICD induction approaches combined with immunotherapy are reviewed. Strategies for engineering NDDSs to enhance their delivery efficiency and activate the tumor immune microenvironment for improving antitumor effects are illustrated.

## 2 Design of NDDSs to Deliver ICD‐Based Therapeutic Agents

In the last decade, NDDSs derived from multi‐functional nanomaterials‐based delivery carriers combined with immunotherapy have gained increasing popularity.^[^ ^25^ ^]^ A myriad of NDDSs have been developed,^[^ ^26^ ^]^ such as liposomes, polymeric nanoparticles (NPs), micelles, dendrimers, and inorganic NPs (including metallic NPs, metal‐organic frameworks, silica NPs, calcium phosphate, etc.) to deliver therapeutic agents including chemotherapeutic drugs, PSs, photothermal conversion agents and radiosensitizers. NDDSs need to take five cascade steps, abbreviated as “CAPIR,” to successfully and effectively deliver therapeutic agents to tumor cells after intravenous administration: circulation in blood compartments (C), accumulation at tumor sites (A), penetration into deep tumor tissues (P), internalization by tumor cells (I), and release of therapeutic agents in intracellular cavities (R).^[^ ^27^ ^]^ The principles in each step provide great insights into constructing rational NDDSs to boost the immunotherapeutic efficacy.

### 2.1 Circulation in Blood Compartments

An adequate blood circulation duration is essential for NDDSs to deliver the loaded therapeutic agents to tumor cells. The first obstacle for NDDSs in a physiological condition is that they can be sequestered by the mononuclear phagocyte system in a short circulation time.^[^ ^28^ ^]^ Upon intravenous administration, plasma proteins such as serum albumin in the blood may be adsorbed onto the surface of NPs,^[^ ^29^ ^]^ and the formed complex could attach to specific receptors on phagocytes and enter into lysosomes of phagocytes to experience degradation.^[^ ^30^ ^]^ Approaches have been developed in order to prolong the circulation time of NDDSs in the blood. Polyethylene glycol (PEG) modification is the most common method to achieve a longer circulation time.^[^ ^31^ ^]^ A hydrating layer around the surface of NDDSs is formed to prevent protein adsorption and clearance by the mononuclear phagocyte system. PEGylation of liposomal or polymeric NPs can significantly increase the half‐life of drugs.^[^ ^32^ ^]^ Another strategy is to engineer biomimetic NPs via coating cell membranes from red blood cells,^[^ ^33^ ^]^ platelets,^[^ ^34^ ^]^ leukocytes,^[^ ^35^ ^]^ or cancer cells^[^ ^36^ ^]^ on the surface of NDDSs to prepare camouflaged nanoformulations.^[^ ^37^ ^]^ The lifespan of red blood cell membrane with expressed CD47 (an integral membrane protein) can be up to 120 days so the membrane can help maintaining long‐term survival of biomimetic NDDSs during the circulation.^[^ ^38^ ^]^ Based on this mechanism, NDDSs with a surface modified with CD47 can inhibit phagocytic clearance and enhance the delivery efficiency.^[^ ^39^ ^]^

### 2.2 Accumulation at Tumor Sites

Sufficient accumulation of therapeutic agents in NDDSs at tumor sites, rather than other sites, maximizes the therapeutic effect while avoiding cytotoxicity to normal cells.^[^ ^40^ ^]^ In a solid tumor tissue with rapid proliferative cells, unique pathophysiological characteristics including hyper vasculatures and defective vascular architectures impair the lymphatic drainage/recovery system.^[^ ^41^ ^]^ NDDSs at a hydrodynamic size of 10–100 nm preferably accumulate within the tumor tissue, which is known as enhanced permeation and retention (EPR) coined by Maeda and their co‐workers in 1986.^[^ ^42^ ^]^ This EPR effect‐based passive targeting approach has been a gold standard to design nano‐sized delivery carriers.^[^ ^43^ ^]^ Besides, active targeting is another alternative way to enhance uptake in tumor cells as well as reduce resistance of tumor cells to therapeutic agents. Monoclonal antibodies, including trastuzumab, rituximab and cetuximab against molecules HER2, CD20, and epidermal growth factor receptor (EGFR) over‐expressed on tumor cell surfaces, respectively, are commonly used for decoration of NDDSs.^[^ ^44^ ^]^ An aptamer, a stable RNA or DNA sequence with double strands, can specifically bind to biological targets such as A10RNA and AS141.^[^ ^45^ ^]^ Furthermore, folic acid,^[^ ^46^ ^]^ iRGD peptides,^[^ ^47^ ^]^ cRGD peptide,^[^ ^48^ ^]^ and transferrin^[^ ^49^ ^]^ can also be decorated on the surface of nanostructures for active targeting at tumor tissues. Besides, exogenous energy‐triggered targeting methods, such as ultrasound, magnetic field, near infrared (NIR) excitation have been demonstrated to display highly localized deposition of therapeutic agents in tumor tissues and improve the efficacy of imaging diagnosis.^[^ ^50^ ^]^

### 2.3 Penetration into Deep Tumor Tissues

Improvement of NDDSs penetration within the solid tumor tissue and their entry into tumor cells away from blood vessels allows released therapeutic agents to eradicate tumor cells in the core of solid tumors and prevent drug resistance. A high interstitial fluid pressure in the solid tumor similar to the aortic artery due to the abnormal intratumoral vascularity prevents deep flow into the solid tumor. Blocked lymphatic drainage and excessive extracellular matrices are other obstacles for deep penetration of NDDSs.^[^ ^51^ ^]^ A strategy is proposed for vascular normalization to mitigate the issue.^[^ ^52^ ^]^ Anti‐angiogenic agents, such as ramucirumab, sorafenib and bevacizumab, can inhibit the expression of vascular endothelial growth factors to hinder angiogenesis, resulting in improving blood flow perfusion into solid tumors to accelerate penetration of NDDSs.^[^ ^53^ ^]^ Furthermore, extracellular matrix‐dissolving agents such as collagenase can reduce the transport resistance.^[^ ^54^ ^]^ It has been reported that NDDSs at a size around 100 nm are favorable in prolonging their circulation time and enhanced their accumulation in the tumor tissue via the EPR effect, while a size smaller than 30 nm is preferable for promoting deep penetration due to less diffusional hindrance.^[^ ^55^ ^]^ This indicates that size shrinkage could be an encouraging strategy to improve both accumulation and penetration.^[^ ^56^ ^]^ Engineered NDDSs with tumor microenvironment‐responsive peptides (e.g., PLGLAG and GFLG) that are cleavable by matrix metalloproteinase‐2/9 (MMP‐2/9), cathepsin B,^[^ ^57^ ^]^ or acidic rapture groups (poly(amidoamine)) were reported to shrink with a diameter smaller than 30 nm for enhanced diffusion into deep tumors.^[^ ^58^ ^]^

### 2.4 Cellular Internalization

After the above “CAP” steps, NDDSs are ready to enter into cells to exert their therapeutic effects. The main pathways for NDDSs entry into cells are endocytosis and direct cellular entry. The surface charge of NDDSs plays an important role in the endocytosis process.^[^ ^59^ ^]^ A neutral or negative surface charge is beneficial for long circulation while a positive one is preferable for electrostatic interaction with negatively charged cell membrane prompting cellular internalization.^[^ ^60^ ^]^ “Charge‐conversion” is explored to facilitate both circulation and internalization.^[^ ^61^ ^]^ For example, *β*‐carboxylic amides of primary or secondary amines are stable at pH 7.4, and they are hydrolyzed in the tumor acidic microenvironment to generate the corresponding amines and protonated to exhibit a cationic charge.^[^ ^62^ ^]^ Similarly, zwitterionic polymers possess a neutral charge under a physiological condition, but becomes positively charged in pH 6.8 to promote endocytosis by tumor cells.^[^ ^63^ ^]^ Decoration by cell‐penetrating peptides (CPPs) is another promising approach. CPPs can be attached onto NDDSs via succinyl amides^[^ ^64^ ^]^ or enzyme‐responsive peptides such as alanine‐alanine‐asparagine.^[^ ^65^ ^]^ They are inactive during circulation but they become active in the tumor tissue microenvironment to enhance NDDSs internalization.

### 2.5 Intracellular Drug Release

After cellular internalization, NDDSs often fuse into lysosomes with a microenvironment of a low pH (pH 4.5–5.5) and abundant enzymes, which helps degradation of extraneous substances to maintain metabolic homeostasis.^[^ ^66^ ^]^ A strategy for endosomal escape of these NDDSs is to disrupt the degradative effect. For example, the “proton sponge” effect is often employed for polymers including polyethyleneimine (PEI), poly‐amido amines, and imidazole‐containing polymers. After they can absorb protons, a great flux of chloride ions and water into the endosomal compartments leads to eventual endosomal rupture to release the cargos to the cytosol.^[^ ^67^ ^]^ In addition, to maintain rapid proliferation of tumor cells, they rewire their metabolism processes to up‐regulate glucose uptake and convert glucose to lactose. This is known as the “Warburg effect,” which results in physiochemical differences of the tumor microenvironment between cancer and normal cells, such as a low pH, a high level of glutathione (GSH) and rich enzymes.^[^ ^68^ ^]^ The differences can be used to fine tune the release of therapeutic agents from NDDSs.^[^ ^69^ ^]^ Acid‐sensitive bonds in NDDSs including hydrazone, thiopropionate, imine, acetal, and ketone can be cleaved under tumor acidic conditions to responsively release cargos.^[^ ^70^ ^]^ Disulfide, diselenide/ditelluride or thioether/selenide‐containing NDDSs are widely explored for their redox‐responsiveness as these bonds are sensitive to an elevated GSH level in tumor cells.^[^ ^71^ ^]^ Furthermore, many enzymes such as MMP‐2/9 and cathepsin are over‐expressed due to pathophysiological changes during tumor progression.^[^ ^72^ ^]^ This provides an additional method to design enzyme‐sensitive NDDSs that can specifically release drugs inside tumor cells. Cleavable peptides are most widely applied, for example, GPLGIAGQ, GPLGV, and GPLCVRG are MMP‐sensitive;^[^ ^73^ ^]^ valine‐citrulline peptides^[^ ^74^ ^]^ and GFLG peptides^[^ ^75^ ^]^ are degradable by cathepsin B.

**Scheme 1:**
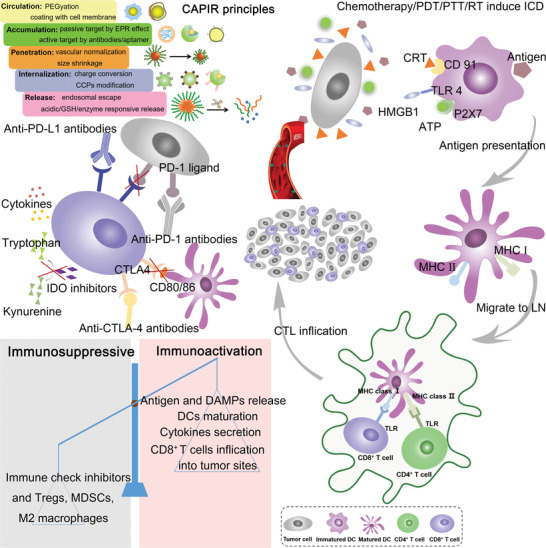
Schematic illustration of “CAPIR” principles for engineering smart NDDSs and the process of chemotherapy/PDT/PTT and RT‐based ICD combined with immunotherapy for cancer therapy. After a rationally designed NDDSs delivers therapeutic agents to tumor cells, the released tumor‐associated antigens can be phagocytosed by DCs and DAMPs (CRT, ATP, and HMGB1) in situ to promote DCs maturation and activate immune responses that reverse the tumor immune microenvironment from immunosuppression to immunoactivation. Furthermore, immunotherapeutic agents such as ICIs can block the PD‐1/PD‐L1 or CTLA‐4/CD28 axis to prevent the immune escape; in turn, the ICD‐inducing immunoresponsive tumor microenvironment can boost the ICIs therapeutic effect.

Through these “CAPIR” principles, elegantly constructed NDDSs can effectively and successfully deliver therapeutic drugs to induce ICD in the tumor site, which is a critical step to activate cancer‐immunity cycle.^[^ ^76^ ^]^ Meanwhile, immunotherapeutic agents may modulate immunosuppressive cells including regulatory T cells (Tregs), myeloid‐derived suppressor cells (MDSCs), M2 macrophages^[^ ^77^ ^]^ and down‐regulate the expression of immunosuppressive molecules such as indoleamine 2,3‐dioxygenase (IDO) to potentiate immunotherapies.^[^ ^78^ ^]^ This is a promising strategy to eradicate neoplasms and inhibit metastasis via acting as vaccination. As shown in **Scheme** **1**, multifunctional NDDSs can be designed from above “CAPIR” principles to mediate ICD and realize immunotherapy, which could become the next‐wave technology for cancer treatment.

## 3 ICD Induced by NDDSs in Combination with Immunotherapy

### 3.1 ICD by Chemotherapy

Chemotherapy is one of the most effective treatments for cancer in clinics. Recent studies reveal that some chemotherapeutics including doxorubicin (DOX), paclitaxel (PTX), and oxaliplatin (OXA) could kill tumor cells via the ICD pathway, which could activate innate and adaptive antitumor immune responses. However, the traditional chemotherapy may cause severe side effects, a high recurrence rate, and a great level of resistance.^[^ ^79^ ^]^ The dissatisfactory effect may be due to a low concentration of these agents in tumor sites and poor penetration into deeper tumor tissues. Chemotherapeutic agents may up‐regulate immunosuppressive molecules such as PD‐L1 and CD 47 to suppress T cells responses.^[^ ^80^ ^]^ Biomaterials‐based smart NDDSs can achieve deep tumor penetration with a changeable size, improve tumor cellular uptake with a switchable surface charge, and avoid side effects with target‐responsive release in tumor cells to promote the ICD effect. Currently, many studies have indicated that ICD inducers loaded in nanocarriers can amplify ICD by releasing more antigens and DAMPs compared to free chemotherapeutic agents.^[^ ^81^ ^]^ These tumor‐associated antigens and DAMPs in situ may enhance the antigenicity and adjuvanticity to induce strong innate and adaptive immune responses, and the antitumor effect can be intensified when chemotherapeutic agents‐based ICD are combined with immunotherapeutic agents. **Table** **1** summarizes the studies of chemotherapies in combination with immunotherapy.

**Table 1:** Chemotherapeutic agents‐medicated ICD combined with immunotherapy

ICD inducer and immunotherapeutic agents	Delivery system	Cytokines or DAMPs	Immune cells infiltration	Model	Ref.
DOX + anti ‐PD‐1 antibodies	Synthetic high‐density lipoprotein (sHDL)‐like nanodiscs	CRT and HMGB1	↑CT26‐specific IFN‐*γ* ^+^CD8a^+^ T cells and neoantigen‐specific CD8a^+^ T cells	CT26 (Balb/c mice) and MC38 (C57BL/6 mice)	^[^ ^6c^ ^]^
DOX + anti‐PD‐1 /anti‐CTLA‐4 antibodies	Liposomes	not available	↑CD8^+^T cells, ↓Tregs	CT26 (Balb/c mice) and MCA205 (C57BL/6 mice)	^[8^ ^1b^ ^]^
DOX + anti ‐PD‐1 antibodies	Nanoprodrug	HMGB1, IFN‐*γ*, TNF‐*α*	↑tumor infiltrating CD4^+^ and CD8^+^ T cells	B16F10 (C57BL/6 mice)	^[^ ^82^ ^]^
DOX + anti ‐PD‐1 antibodies	A murine melanoma cell membrane‐coated biomimetic nanoplatform based on zeolitic imidazolate framework 8	IFN‐*γ*, TNF‐*α*, IL‐2, IL‐6	↑CD8^+^ T cells recruited to tumor	B16F10 (C57BL/6 mice)	^[^ ^83^ ^]^
DOX + anti ‐PD‐1 antibodies	Dendritic mesoporous silica materials‐based nanoreactors that are capable of triggering the Fenton reaction and GSH depletion	CRT, TNF‐*α*, IL‐12, and IL‐1	↑infiltration of CTLs in the distant tumors	4T1 (Balb/c mice)	^[^ ^84^ ^]^
DOX + monophosphoryl lipid A (MPLA) + anti‐PD‐L1 antibodies	DSPE‐PEG‐based micelles	CRT, ATP and HMGB1, IFN‐*γ*, TNF‐*α*, IL‐2	↑DCs maturation and MHC II expression on the surface of CD11c^+^ DCs ↑infiltrating CD8^+^ or CD4^+^ T cells and ↓Tregs	B16F10 (C57BL/6 mice)	^[^ ^85^ ^]^
PTX + anti‐PD‐1 antibodies	Micelles from a copolymer of azide‐terminated PEG and polyaspartic acid	CRT, IFN‐*γ*, TNF‐*α*	Increased tumor infiltrated CD8^+^ T cells	B16F10 (C57BL/6 mice)	^[^ ^86^ ^]^
Digitoxin + carboplatin + siRNA against PD‐L1	Acidic sensitive polymer NPs	CRT, ATP, HMGB1, HSP 70, IFN‐*γ*	↑APCs and the ratio of M1/M2 macrophage; ↑CD3*ε* ^+^CD4^+^ helper T cells and CD3*ε* ^+^CD8a^+^ cytotoxic T cells, ↓Tregs	CT26 and MC38 (Balb/c and C57BL/6 mice)	^[^ ^87^ ^]^
Epirubicin + PD‐L1 blockaded antibodies	*N*‐(2‐hydroxypropyl) methacrylamide NPs	CRT, HMGB1	↑DCs maturation and the ratio of CD8^+^ T cells to Tregs ↑CD8^+^ T cell/ Tregs ratio	4T1 (Balb/c mice)	^[^ ^88^ ^]^
Shikonin + JQ1	Mannosylated lactoferrin NPs	CRT, HMGB1	↑DCs maturation, CD8^+^ T cell infiltration and M1 macrophages ↓Tregs and M2 macrophages	CT26 (Balb/c mice)	^[^ ^89^ ^]^
OXA + DHA + anti‐PD‐L1 antibodies	Nanoscale coordination polymeric core‐shell particles	CRT, HMGB1	↑infiltration of DCs and macrophages in tumors ↑the percentage of M1 macrophages and the density of CD8^+^ T cells	CT26 (Balb/c mice) and MC38 (C57BL/6 mice)	^[^ ^90^ ^]^
17‐(allylamino)‐17‐demethoxygeldanamycin (17‐AAG) + anti‐PD‐L1 antibodies	Liposomes	CRT, HMGB1	↑activated DCs population and tenfold increase infiltrating T cells in tumor ↓M2 macrophages and MDSCs	4T1 (Balb/c mice)	^[^ ^91^ ^]^
DOX + IFN‐*γ*	Thermosensitive NPs	TGF‐*β*, IL‐10, TNF‐*α* and IL‐2	↑DCs maturation, CTLs infiltration, and the number of NK cells	B16F10 (C57BL/6 mice)	^[^ ^92^ ^]^
PTX +IL‐2	Biomimetic nanogel from hydroxypropyl‐*β*‐cyclodextrin acrylate and chitosan derivatives	CRT, IFN‐*γ* and IL‐12	↑CD8^+^ T cells infiltration, NK cell and DCs maturation ↓Tregs	B16F10 (C57BL/6 mice)	^[^ ^93^ ^]^
PTX +IL‐2	Thermosponge NPs formulated by poly(lactic‐co‐glycolic‐acid (PLGA) and Pluronic F127	IL‐10, IL‐12 and IFN‐*γ*	↑DCs infiltration and promoted DCs maturation ↑CD3^+^CD8^+^ T cells and CD3^+^CD4^+^ T cells, ↓Tregs	B16F10 (C57BL/6 mice)	^[^ ^94^ ^]^
DOX + CpG	MnO_2_ nanosheet functioned as a unique support to integrate DOX and Ag‐CpG and Ag nanoclusters for increasing CpG stability against nuclease degradation	CRT, ATP and HMGB1, IL‐6, TNF‐*α*	↑CD8^+^ cytotoxic T lymphocytes and CD4^+^ T cells ↓ Tregs	4T1 (Balb/c mice)	^[^ ^95^ ^]^
Mitoxantrone + CpG	A lipid‐polymer hybrid nanodepot platform	HMGB1	↑IFN‐*γ* ^+^ CD8*α* ^+^ and CD4^+^ T cells	CT26 (Balb/c mice)	^[^ ^96^ ^]^
DOX + CpG	Not available	Not available	↑CD8^+^ T cells infiltration	CT26 (Balb/c mice)	^[^ ^97^ ^]^
DOX + siRNA	A carrier‐free nanoassembly with PEG as a shell and DOX/siRNA as a core	CRT, ATP and HMGB1	↑CD8^+^ T cells and IFN‐*γ*, ↓PD‐L1 expression	CT26 (Balb/c mice)	^[^ ^98^ ^]^
DOX + siRNA	Cationic polymer‐lipid hybrid nanovesicles	CRT, ATP and HMGB1	↑CD4/8^+^T cells and DCs maturation	B16F10 (C57BL/6 mice)	^[^ ^99^ ^]^
DOX + MicroRNA	Folic acid conjugated PLGA‐PEG and PLGA‐PEI NPs	CRT and HMGB1	↑CD8^+^ T cells, DCs maturation and IFN‐*γ*, TNF‐*α* secretion ↓PD‐L1 expression	MC38 (C57BL/6 mice)	^[^ ^100^ ^]^

#### 3.1.1 Chemotherapy Coupled with Immune Checkpoint Inhibitors (ICIs)

A low patient response rate during ICIs therapy mostly ascribes to the absence of tumor T cells infiltration. ICD could promote DCs maturation and increase tumor‐infiltrating lymphoid and myeloid cells,^[^ ^101^ ^]^ which create an immunoresponsive tumor microenvironment.^[^ ^77^ ^]^ By displaying a high degree of T cells infiltration, hot tumors become a fertile ground for effective ICIs‐based therapies.^[^ ^102^ ^]^ It has been demonstrated that a hot tumor microenvironment with infiltrated CTLs can significantly increase the response rate to anti‐PD‐1/PD‐L1 antibodies.^[^ ^103^ ^]^ Therefore, the combination of ICD and ICIs can dramatically improve the antitumor effect.^[^ ^104^ ^]^ Recently, this combinational therapy has been extensively studied in both preclinical and clinical phases, indicating its great prospect in regression of solid tumors.

The anthraquinone like DOX can prevent DNA replication and RNA synthesis and mediate subapoptotic activation of capase‐8. It has been confirmed to induce ICD and it has been widely used as an ICD inducer. Rios‐Doria et al. reported that liposomal doxorubicin (Doxil) boosted the antitumor responses after Doxil was combined with several different immune checkpoint blockers including anti‐PD‐1/PD‐L1, CTLA‐4 monoclonal antibodies and TNF receptor agonists. This study indicated that Doxil treatment increased CD8^+^ T cells infiltration and simultaneously decreased the ratio of Tregs in tumors, suggesting that Doxil could play a novel immunomodulation role in chemotherapy.^[^ ^81b^ ^]^ This study has shown a great potential for clinical translation of this treatment method. In addition, the combinational therapy could reduce DOX dosage and reduced its related toxicities. Similar therapeutic effects were reported by Kuai et al. They employed high‐density lipoprotein (sHDL)‐like nanodiscs as a delivery system, composed of apolipoprotein A1 (ApoA1) mimetic peptides and phospholipids, to stimuli‐responsively release DOX. The nanodiscs released DOX in the acidic endosomes of tumor cells to avoid the off‐target side effects.^[^ ^6c^ ^]^ ICD was successfully induced by released DOX from sHDL‐DOX, which was evidenced with a high level of CRT expressed on the tumor cell surface and robustly released HMGB1.Thus, the frequency of IFN‐*γ* ^+^ CD8a^+^ T cells in CT26 tumor‐bearing mice was sevenfold higher than that in the free DOX treatment group. In this immune‐activated microenvironment, anti‐PD‐1 antibodies could eventually regress up to 80–88% of established CT26 colon carcinoma tumors as well as prevent tumor recurrence and metastasis in the liver.^[^ ^6c^ ^]^ Analogously, Gao et al. and Zou et al. also showed the co‐treatment of DOX with anti‐PD‐1 antibodies leading to very promising tumor regression effects in the B16F10 tumor mice model.^[^ ^82^, ^83^, ^84^ ^]^ In particular, tumor cell membranes‐coated biomimetic nanoparticles, which were composed of pH‐sensitive zeolitic imidazolate framework 8 for co‐delivery of DOX and catalase, were able to escape from the immune system and target tumor cells through their adhesion proteins. Catalase in the core of the nanoplatform decomposed H_2_O_2_ to produce O_2_ in the tumor microenvironment to relieve hypoxia, directly reducing hypoxia‐inducible factor‐1*α* (HIF‐1*α*) expression and indirectly down‐regulating the expression of PD‐L1. Furthermore, combination of DOX‐induced ICD for increasing specific T cells in the tumor microenvironment and anti‐PD‐1 antibodies dually blocked the PD‐1/PD‐L1 immune escape axis, which indicated that the multifunctional nanoplatform with tumor microenvironment‐mediation ability could contribute to the synergic antitumor effect.^[^ ^83^ ^]^ In addition, DOX and siRNA such as miR‐200c were combined to down‐regulate the expression of PD‐L1 in tumor cells, exhibiting excellent tumor growth inhibition whilst promoting antitumor immune responses.^[^ ^98^, ^99^, ^100^ ^]^

**Figure 1:**
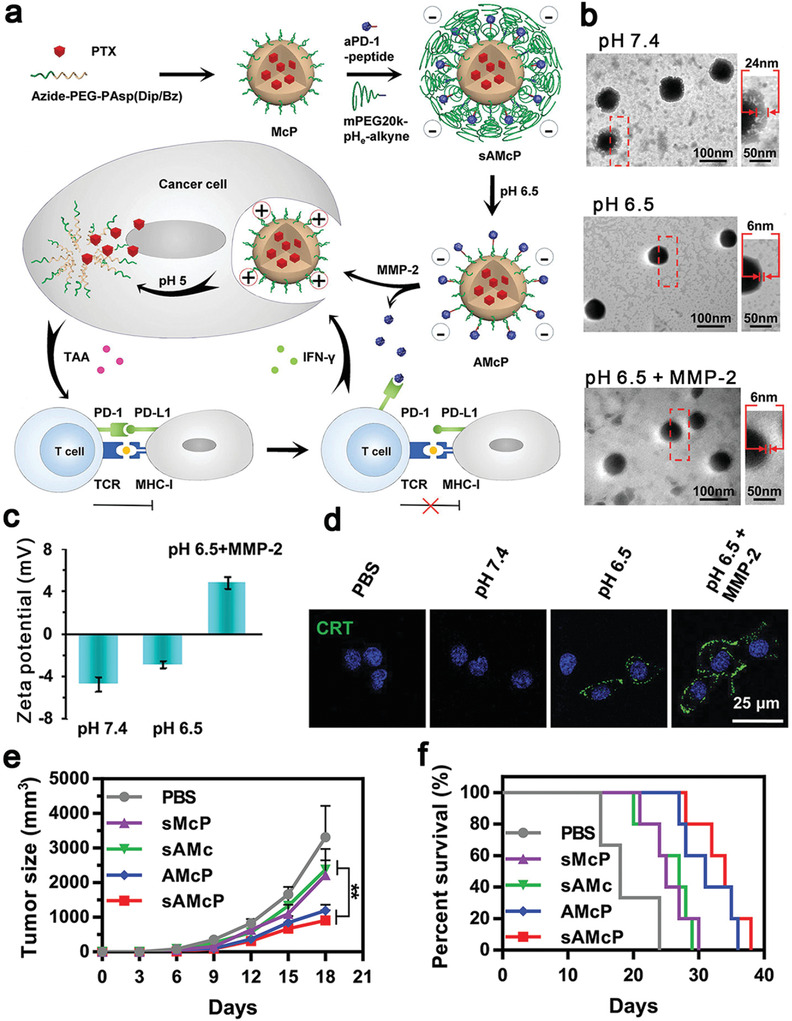
a) Schematic illustration of dual pH and MMP‐2 responsive micelles (sAMcP) to load both PTX and anti‐PD‐1 antibodies. b) TEM images of sAMcP after treatment at pH 7.4, pH 6.5, and pH 6.5 in the presence of 1 × 10^−8^ m MMP‐2 to shed the PEG layer and responsively release anti‐PD‐1 antibodies in the tumor microenvironment. c) Charge switchable property of sAMcP at pH 7.4, 6.5, and 6.5 in the presence of 10 × 10^−9^ m MMP‐2. d) Confocal images of CRT released on tumor cells incubated with sAMcP under different pH values. Antitumor effects on B16F10 tumor‐bearing mice: e) tumor growth curves and f) survival rates. Reproduced with permission.^[^ ^86^ ^]^ Copyright 2020, Wiley‐VCH.

Another chemotherapeutic agent, PTX, has also been demonstrated to act as an ICD inducer. For example, Su et al. engineered a pH and MMP dual‐sensitive micellar nanocarrier (sAMcP) to load both PTX and anti‐PD‐1 antibodies (**Figure** **1**a). sAMcPs were obtained from assembly of a copolymer of azide‐terminated polyethylene glycol/polyaspartic acid. During the assembly process, PTX was encapsulated within the core of sAMcPs. Anti‐PD‐1antibodies were decorated on the shell of the micelle via MMP‐2 sensitive peptide fragments. Cleavable long‐chain PEG (PEG20k) was coated on the micellar nanostructure via azide and alkyne click reaction.^[^ ^86^ ^]^ This delivery system was responsive in the tumor microenvironment with a low pH and abundant MMP‐2 and showed spatio‐temporally controlled release of drugs. First, the outer layer of PEG was peeled from the delivery system in pH 6.5 (Figure 1b), resulting in a switch from negative charge to positive one (from −2.9 ± 0.3 mV to +4.8 ± 0.6 mV) (Figure 1c) for facilitating the internalization of micelles into cancer cells. Meanwhile, anti‐PD‐1 antibodies were released in the tumor microenvironment in response to MMP‐2. Finally, the pH sensitive core was responsive to lysosomal acidity (pH ≈ 5.0) to release PTX. The on‐demand released PTX induced CRT exposure on the cell membrane (Figure 1d) to boost the infiltration of CD8^+^ T cells, while anti‐PD‐1 antibodies blocked the PD‐1/PD‐L1 axis to suppress the immune escape to intensify the ICD effect, leading to a remarkable antitumor chemoimmunotherapeutic efficacy in a murine melanoma model (Figure 1e,f).

**Figure 2:**
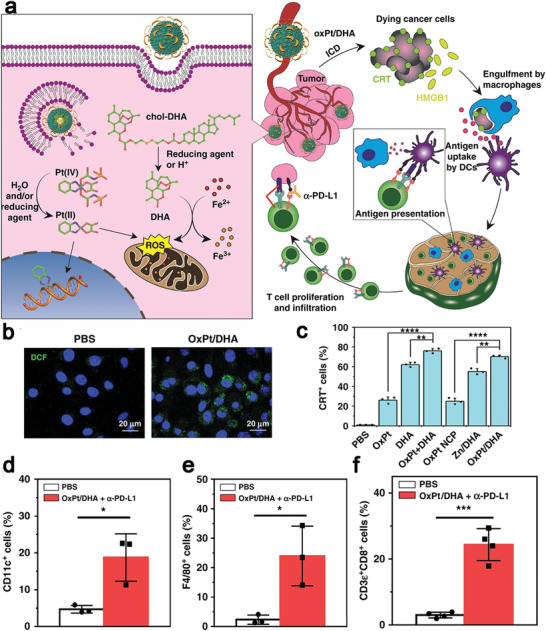
a) Illustration of nanoscale coordination polymeric core‐shell particles loaded with DHA and OxPt to induce ICD for cancer treatment. DHA and OXA were responsively released in the presence of abundant GSH in a tumor physiological condition. b) ROS generation ability in CT26 cells after OxPt/DHA treatment. c) Flow cytometry analysis of CRT exposure on the surface of tumor cells. Treatment with OxPt/DHA + anti‐PD‐L1 antibodies (*α*PD‐L1) remarkably enhanced d) DCs (CD11c^+^) and e) macrophages (F4/80^+^) infiltration into tumors for antigens presentation, and facilitated migration of f) CD8^+^ T cells (CD3*ε* ^+^ CD8^+^) in tumors for adaptive immune responses. Reproduced with permission.^[^ ^90^ ^]^ Copyright 2019, Springer Nature.

As a first‐line candidate to treat colorectal cancer, oxaliplatin (OXA) triggers immunogenic apoptosis and CRT exposure and it is also a potent ICD inducer.^[^ ^105^ ^]^ Duan et al. constructed core–shell NPs from a self‐assembled nanoscale coordination polymer, loaded the OXA prodrug (OxPt) within its core and cholesterol‐conjugated dihydroartemesinin (DHA) in the shell (**Figure** **2**a).^[^ ^90^ ^]^ DHA, a reactive oxygen species (ROS)‐producing drug, was used to complement the OXA‐based ICD effect due to its ability to trigger mitochondrial dysfunction promoting apoptosis and also induce the ER stress (Figure 2b). OxPt/DHA NPs at a size of 73.8–103.4 nm and the negative surface charge not only avoided uptake by the mononuclear phagocyte system to achieve selective accumulation in the tumor tissue, but also protected the DHA stability under a physiological condition. Treatment with OxPt/DHA NPs enhanced the CRT exposure ratio on tumor cells (Figure 2c), which increased infiltration of APCs (e.g., DCs and macrophages (Figure 2d,e)) into tumor sites to capture and present antigens. Moreover, the density of CTLs in tumors (Figure 2f) was elevated to create an immunoresponsive microenvironment that strengthened the anti‐PD‐L1 antibodies therapeutic effect. In the mice co‐treated with OxPt/DHA and anti‐PD‐L1 antibodies, 6/6 tumors were eventually regressed and a memory response was induced, resulting in immunity against subsequent challenges by live tumor cells. The study demonstrated the ICD effect can reshape the immunosuppressive microenvironment to an immunostimulative one through innate and adaptive immune systems, and ICD in combination with anti‐PD‐L1 antibodies enhanced the tumor eradication efficiency.^[^ ^90^ ^]^ In addition, other ICD‐based chemotherapeutic agents, such as digitoxin,^[^ ^87^ ^]^ epirubicin,^[^ ^88^ ^]^ shikonin,^[^ ^89^ ^]^ and 17‐(allylamino)‐17‐demethoxygeldanamycin^[^ ^91^ ^]^ have been reported to trigger tumor‐specific immune responses, which were further augmented by blocking the PD‐1/PD‐L1 pathway to reestablish immune surveillance to remove re‐challenged tumor cells so that long and lasting immune protection was realized.

#### 3.1.2 Chemotherapy Combined with Cytokines

Cytokines play a critical role in the innate and adaptive immune system. Paracrine or autocrine cytokines released from cells are small proteins with a molecular weight range of 5–20 kDa. They act as immunomodulating agents to control proliferation and differentiation of immune cells, and perform functions to further enhance their antitumor activity through propagation of immune signals and trigger of immune responses.^[^ ^106^ ^]^ Cytokines‐based immunotherapy possesses an enormous potential for cancer eradication in multiple aspects.^[^ ^107^ ^]^ Several cytokines have been approved by the Food and Drug Administration (FDA). The first marketed immunotherapeutic cytokine is IFN‐*α* for hairy cell leukemia which was approved in 1986.^[^ ^1a^ ^]^ Subsequently, IL‐2 was approved for renal cancer and metastatic melanoma.^[^ ^108^ ^]^ The ICD effect triggered by chemotherapy could be strengthened in combination with cytokines‐based immunotherapy, thus, potent immune responses were elicited to promote maturation of DCs /NK cells and increase the number of CTLs in the tumor environment,^[^ ^109^ ^]^ which has been demonstrated to be a promising approach against melanoma.^[^ ^110^ ^]^

**Figure 3:**
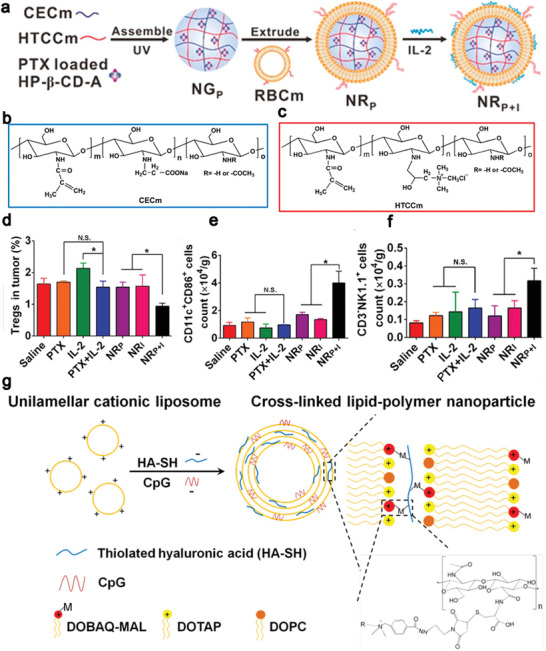
Chemotherapy combined with cytokines and adjuvants. a–c) Schematic illustration of the preparation process to engineer an erythrocyte membrane‐coated nanogel (NR) for co‐delivery of PTX and IL‐2. Chemical structures of a) chitosan derivatives of amphoteric methacrylamide *N*‐carboxyethyl chitosan (CECm) and b) positively charged methacrylamide N‐(2‐hydroxy)propyl‐3‐trimethylammonium chitosan chloride (HTCCm). Immune cells in the tumor microenvironment after different treatments, d) reducing the ratio of Tregs and increasing infiltration of immune effector cells such as e) mature DCs, f) NK cells. Reproduced with permission.^[^ ^93^ ^]^ Copyright 2017, American Chemical Society. g) Preparation of cross‐linked lipid‐polymer NPs between anionic thiolated hyaluronic acid (HA‐SH) and cationic maleimide‐modified unilamellar liposomes via charge‐mediated complexation to load CpG. Reproduced with permission.^[^ ^96^ ^]^ Copyright 2017, American Chemical Society.

To co‐deliver small molecular chemotherapeutic drugs and biomacromolecular cytokines that have distinctive differences in physicochemical properties, nanocarriers must have multiple functions to achieve a high load efficiency and maintain the bioactivity of biomacromolecules. To address the dilemma, Yin et al. employed a copolymer, PLGA and PEO‐PPO‐PEO (pluronic F127), to construct thermosensitive nanoparticles (TSNs). DOX was conjugated onto PLGA via a pH sensitive hydrazone bond as a core of TSNs and IFN‐*γ* was adsorbed onto the shell of TSNs via electrostatic interaction.^[^ ^92^ ^]^ In this delivery system, the size of TSNs was adjustable in response to temperature and it decreased from 100.5 ± 6.3 nm at 25 °C to 89.5 ± 4.0 nm at 37 °C, achieving excellent accumulation in the tumor site in vivo via the EPR effect. On the other hand, DOX was released in an intracellular pH‐triggered sustained manner for up to 7 days, resulting in sustained antitumor response by inducing ICD. TSNs protected the bioactivity of IFN‐*γ* and avoided IFN‐*γ* leakage in the peripheral tissue. Taken together, DOX/IFN‐*γ* TSNs promoted DCs maturation, CTLs infiltration and NK cells proliferation in the tumor environment to provoke innate and specific antitumor immune responses resulting in a tumor inhibitory rate of 88.4 ± 1.7%. Similar results were reported by Song et al. who developed a biomimetic nanogel from two oppositely charged chitosan derivatives (amphoteric methacrylamide *N*‐carboxyethyl chitosan and positive charged methacrylamide *N*‐(2‐hydroxy)propyl‐3‐trimethylammonium chitosan chloride) and 2‐hydroxypropyl‐*β*‐cyclodextrin acrylate. The nanogel was loaded with PTX and IL‐2 and coated with erythrocyte membranes to prolong its circulation time (**Figure** **3**a–c). First, PTX was released in response to a low pH in the tumor microenvironment to induce ICD, leading to reducing the ratio of Tregs in tumors (Figure 3d) and increasing CRT exposure on the tumor cell surface to promote immature DCs to phagocytose the tumor cell debris. On the other hand, the released IL‐2 enhanced DCs maturation and NK cells activation (Figure 3e,f). The nanogel‐mediated chemo‐immunotherapy induced CD8^+^ T cell immune responses in B16F10 tumor bearing mice, leading to remarkable inhibition of tumor growth.^[^ ^93^ ^]^ The study indicated that the nanogel could be an effective delivery system to co‐encapsulate both hydrophilic and hydrophobic drugs. These smart NDDSs for co‐delivery of an ICD inducer and cytokines (e.g., IFN‐*γ* or IL‐2) could further improve the antitumor effect by stimulating the immune system. IFN‐*γ* could promote T cells differentiate to tumor‐specific CD4^+^ T cells and expansion of CTLs, while IL‐2 promoted activation and proliferation of NK cells.^[^ ^111^ ^]^

#### 3.1.3 Chemotherapy Combined with Immunoadjuvants

During ICD, tumor cells could release neoantigens and danger signals that can be captured by DCs and macrophages to further present to immune cells, leading to special tumor antigens‐induced immune responses.^[^ ^112^ ^]^ For this reason, ICD have been proposed as potential vaccination in situ without sophisticated procedures and ethical concerns compared with traditional vaccines.^[^ ^113^ ^]^ Unmethylated cytosine‐phosphate‐guanine (CpG), an immunostimulatory nucleic acid, is a powerful toll‐like receptor‐9 (TLR9) agonist to induce a cascade of adaptive and innate immune responses, and it has been widely used as an adjuvant.^[^ ^114^ ^]^ CpG can specifically bind to TLR9 to induce the production of cytokines such as IFN‐*γ*, IL‐12, IL‐6, TNF‐*α*, and increase the level of co‐stimulatory molecules expressed by B cells and DCs.^[^ ^115^ ^]^ In particular, clinical trial results have indicated that CpG can enhance T cells responses to boost the antitumor effect when it is used as a single agent or it is combined with antineoplastic therapies.^[^ ^116^ ^]^ Therefore, CpG combined with ICD‐based therapeutic agents may have a potent efficacy against cancer cells as CpG can intensify the adjuvanticity of ICD to establish vaccination.

An MnO_2_ nanosheet‐based delivery system to co‐load CpG‐silver nanoclusters and DOX showed excellent antitumor efficiency in 4T1 tumor‐bearing mice. When the nanocomposites were internalized by tumor cells, DOX promoted tumor cells to secrete CRT, HMGB1 and ATP as danger signals to stimulate antitumor immune responses, while CpG acted as a powerful promoter. This treatment exhibited admirable antitumor immunity by increasing proliferation of CD4^+^ T cells and the number of tumor infiltrating CD8^+^ T cells, resulting in a 95% inhibition rate for 4T1 tumor cells.^[^ ^95^ ^]^ Fan et al. demonstrated excellent therapeutic outcomes for melanoma and colon carcinoma murine tumors by converting immunogenic dying tumor cells into a potent cancer vaccine in two steps.^[^ ^96^ ^]^ In brief, they incubated colon carcinoma CT26 and melanoma B16 cells with mitoxantrone to induce ICD to release antigens and HMGB1, and then the dying tumor cells were incubated with nano‐multilayered particles containing CpG to amplify the adjuvant potency (Figure 3g). The nano‐multilayered particles were engineered with maleimide‐modified lipid, which was specifically conjugated with sulfhydryl on the surfaces of died tumor cells via thiol‐maleimide chemistry. The surface of dying tumor cells after conjugation with CpG‐NPs displayed great promotion in DCs maturation and cytokines (IL‐12 and TNF‐*α*) secretion compared with a physical mixture of free CpG and dying tumor cells. The designed vaccine efficiently promoted antigens cross‐presentation by DCs and triggered antigen‐specific T cells responses with an enhanced antitumor activity in a colon carcinoma mice model. To directly induce vaccination in situ, Walters et al. replaced the modification process ex vivo to simplify the vaccination process. They injected the ICD inducer DOX into a solid tumor to generate immunogenic apoptotic fragments or debris, then CpG conjugated with cholesterol was injected into the tumor which specifically targeted the tumor cell membrane.^[^ ^97^ ^]^ In vivo studies revealed that the synergistic treatment delayed tumor growth and elevated the level of CD4^+^ and CD8^+^ T cells in CT26 tumor‐bearing mice. These studies indicated that CpG as an adjuvant can promote the therapeutic effect of chemotherapy‐based ICD by increasing the number of tumor infiltrating lymphocytes, and multiple NDDSs can be employed to boost the ICD effect. Other adjuvants, such as resiquimod (R848), a TLR7/8 agonist, can synergize with DOX to form the in situ vaccination, resulting in sixfolds of CD8^+^ T cells in the tumor microenvironment and a regression rate of 40% against 4T1 tumors.^[^ ^117^ ^]^

ICD induced by chemotherapeutic agents in combination with ICIs, cytokines and immunoadjuvants has been demonstrated with a remarkable antitumor efficacy. Furthermore, chemotherapeutic agents combined with IDO inhibitors to modulate the tumor microenvironment, such as indoximod,^[^ ^118^ ^]^ NLG919,^[^ ^119^ ^]^ 1‐methyl‐tryptophan,^[^ ^120^ ^]^ and small interfering RNAs^[^ ^121^ ^]^ have also been demonstrated to inhibit tumor growth and prevent the metastasis. Therefore, combined chemotherapy‐based ICD with immunotherapies may open a new avenue for solid cancer treatment.

### 3.2 ICD by Photodynamic Therapy

PDT is currently used as an alternative treatment to control malignant tumors.^[^ ^122^ ^]^ PDT can destroy tumor cells and diseased tissues through cytotoxic ROS including hydrogen peroxide, hydroxyl radicals, superoxide anion, and singlet oxygen. These ROS are often generated from three key components: light, oxygen, and PSs.^[^ ^123^ ^]^ There are two steps for PSs to generate cytotoxic ROS. First, after light irradiation, the PS could convert to an unstable singlet state, which could directly transfer energy to ground‐state triplet oxygen (^3^O_2_) to generate highly reactive singlet oxygen (^1^O_2_), or react with a biomolecule O_2_ or H_2_O to produce ROS.^[^ ^124^ ^]^ Under oxidative stress, damaged tumor cells elicit ICD to release DAMPs and neoantigens to activate innate and adaptive immune responses. Furthermore, PDT can lead to rapid recruitment of various immune cells to tumor sites to activate the tumor immune microenvironment and secrete various cytokines (such as IL‐6, IL‐1*β*, and TNF‐*α*) to intensify immune responses.^[^ ^125^ ^]^ Although PDT triggers the antitumor immunity, its effect is relatively weak and transient.^[^ ^2b^ ^]^ Many studies have shown that intense PDT results in up‐regulation of immunosuppressive molecules such as PD‐1, CTLA‐4, and IDO to mediate immune evasion.^[^ ^126^ ^]^ Therefore, PDT combined with ICIs such as anti‐PD‐1/PD‐L1 antibodies to block the PD‐1/PD‐L1 pathway or IDO inhibitors to modulate the tumor microenvironment could be a potential approach to amplify the immunity induced by PDT for cancer treatment (**Table** **2**).

**Table 2:** PDT/PTT triggered ICD in combination with immunotherapies

ICD inducers and immunotherapeutic agents	Delivery system	DAMPs	Advantages	Model	Ref.
Based on PDT					
Photosensitizer pyrolipid + anti‐PD‐L1 antibodies	Zn‐pyrophosphate NPs	CRT	Increasing the solubility of hydrophobic photosensitizer, prolonging blood circulation and excellent biocompatibility	4T1 and TUBO murine breast cancer (Balb/c mice)	^[^ ^127^ ^]^
IR780 + anti‐PD‐L1 peptide	NPs	CRT	Self‐assembly, high loading efficiency and MMP‐2 responsiveness to transit into a smaller size for deep tumor penetration	B16F10 (C57BL/6)	^[^ ^129^ ^]^
Nanophotosensitizer (Fe‐TBP) + anti‐PD‐L1 antibodies	nMOFs	CRT	Overcoming tumor hypoxia	Bilateral CT26 (Balb/c mice)	^[^ ^132^ ^]^
Ppa + JQ1	Supramolecular prodrug nanoplatform	CRT, HMGB1	JQ1 down‐regulated the expression of c‐Myc and PD‐L1 to modify the hypoxia‐mediated immunosuppressive microenvironment; JQ1 combined with PDT induced intense memory immune responses for inhibition of lung metastasis	Panc02 cell (C57BL/6)	^[^ ^136^ ^]^
PEGylated Ppa + reduction‐sensitive IDO inhibitor (NLG919)	Prodrug vesicle	CRT, HMGB1	Keeping stable in the bloodstream, avoiding drug leakage, GSH‐responsive release of NLG919, MMP‐2 responsive to shed the PEG corona for deep tumor penetration	CT26 and 4T1 (Balb/c mice)	^[^ ^140^ ^]^
HPPH + IDO inhibitor indoximod	pH‐responsive nanovesicles	CRT	Self‐assembly, carriers as an inducer, achieving endosomal escape to release cargos in the cytoplasm	B16F10 (C57BL/6)	^[^ ^141^ ^]^
Photosensitizer chlorin e6 (Ce6) + honey bee venom melittin (MLT) peptide + anti‐PD‐1 antibodies	Organic–inorganic nanocarrier	CRT, ATP	Producing ROS by Ce6‐based PDT to induce ICD and activating DCs by MLT to enhance the ICD effect	4T1(Balb/c mice)	^[^ ^160^ ^]^
Ppa + IDO inhibitor (NLG919)	Redox‐activatable liposome	CRT, HMGB1 and ATP	Self‐assembly, prolonging blood circulation, responsively releasing cargos at a high level of GSH in tumors	4T1 (Balb/c mice)	^[^ ^161^ ^]^
Chlorin derivative + IDO inhibitor (INCB24360)	Chlorin‐based nMOFs	CRT	High PS loading efficiency, facilitating intersystem interactions to enhance ^1^O_2_ generation	CT26 (Balb/c mice) and MC38(C57BL/6)	^[^ ^162^ ^]^
Photosensitizer PpIX + IDO inhibitor (1MT)	Chimeric peptide NPs	CRT	Self‐assembly, releasing 1MT in response to caspase‐3	CT26 (Balb/c mice)	^[^ ^163^ ^]^
Based on PTT					
Single‐walled carbon nanotubes + anti‐CTLA‐4 antibodies	Nanotubes	Not available	Greatly promoting maturation of DCs and producing antitumor cytokines	4T1 (Balb/c mice)	^[^ ^147^ ^]^
Black phosphorus nanosheets + CpG	Nanosheets	CRT, HMGB1 and ATP	Excellent physicochemical characteristics, biodegradability, and biocompatibility	4T1 (Balb/c mice)	^[^ ^149^ ^]^
Gold NPs + imiquimod + anti‐ PD‐1 blockade	2D polypyrrole nanosheets (PPy) and PEG‐PLGA micellar NPs	CRT, HMGB1 and ATP	Inducing DAMPs release in deeper tumor sites	4T1 (Balb/c mice)	^[^ ^151^ ^]^
Cu‐containing layered double hydroxide + HSPs inhibitor	Nanohybrid	CRT	Triggering sufficient cytotoxic ROS in cancer cells by Fenton reaction to amplify ICD	4T1 (Balb/c mice)	^[^ ^152^ ^]^
Hollow gold nanoshells + anti‐PD‐1 peptide (AUNP12) + CpG	PLGA NPs	HSP 70	Maintaining release up to 40 days for the peptide	4T1 (Balb/c mice) and CT26(C57BL/6)	^[^ ^164^ ^]^
Prussian blue (PB) + Sorafenib + anti‐ PD‐L1 antibodies	PB NPs	CRT, ATP and HMGB1	PB NPs serve as nanocarriers, photothermal conversion agents, and imaging agents	Hepa1−6 (C57BL/6)	^[^ ^154^ ^]^
Copper sulfide (CuS) NPs + resiquimod (R848) + AUNP‐12	Dendritic large‐poremesoporous silica nanoparticles (DLMSNs)	CRT, Hsp 70	Increasing the tumor targeting ability by coating cancer membrane, releasing R848 and AUNP‐12 via cleavage of an acid‐labile benzoic‐imine bond, inducing apoptosis by CuS based PTT	4T1 (Balb/c mice)	^[^ ^155^ ^]^
Semiconducting polymer + 4T1 cell membrane + DCs membrane	NPs	HMGB1	SPN with a photothermal conversion efficiency of 88.8% that were coated with pre‐engineered 4T1 cell membrane treated with DOX and DCs membrane stimulated with resiquimod served as a nanovaccine to ablate tumors	4T1 (Balb/c mice)	^[^ ^158^ ^]^
Semiconducting polymer + resiquimod	Thermally responsive lipid NPs	CRT, ATP, HMGB1	Resiquimod that was thermal‐responsive released when the temperature rose to 42 °C was combined with PTT‐mediated ICD effectively inhibited primary and distant tumor growth	4T1 (Balb/c mice)	^[^ ^159^ ^]^

#### 3.2.1 PDT in Combination with ICIs

**Figure 4:**
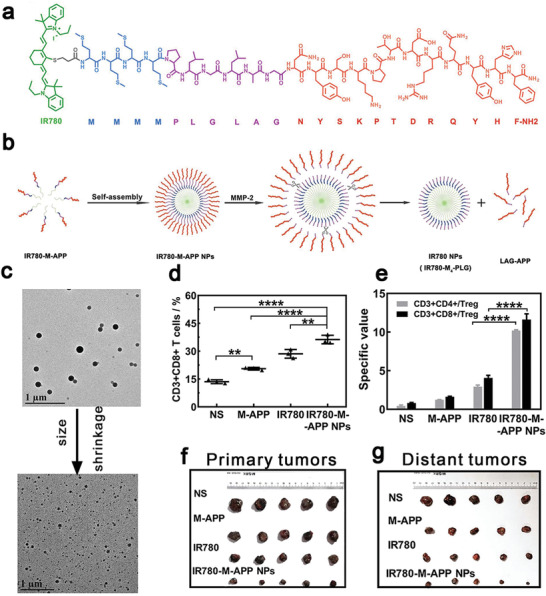
a) IR780 (green) was connected with hydrophilic APP (red) by short methionine fragments (blue) and MMP‐2 responsive cleaved linkers (PLGLAG: purple). b) Schematic illustration of the self‐assembly process to construct IP780‐M‐APP NPs. c) TEM images of IP780‐M‐APP NPs and smaller particles after incubation with MMP‐2. After intravenous injection, d) PDT increased the number of CD3^+^CD8^+^ T cells, e) while down‐regulated the ratio of Tregs. IR780‐M‐APP NPs killed both f) primary and g) distant tumors. Reproduced with permission from.^[^ ^129^ ^]^ Copyright 2020, Elsevier.

PDT can initiate antitumor immune responses to improve the sensitivity of the checkpoint therapy, therefore, it can be combined with anti‐PD‐1/PD‐L1 antibodies or peptides to inhibit both primary and distant tumors. Non‐toxic Zn‐pyrophosphate (ZnP) NPs loaded a photosensitizer pyrolipid (ZnP@pyro), which increased the solubility of the photosensitizer and prolonged its blood circulation time (14.5 ± 2.2 h) to enhance the effect of PDT.^[^ ^127^ ^]^ At a size of 45.4 ± 2.8 nm, NPs were preferential to accumulate in tumor tissues due to the EPR effect to induce a high level of CRT exposure compared with free pyrolipid. During this ZnP@pyro‐based PDT and PD‐L1 checkpoint blockade combined therapy process, systemic tumor‐specific CTLs responses were generated to directly eradicate primary tumors and completely inhibit untreated distant tumors, and the antitumor immunity was activated to effectively prevent lung metastases in the 4T1 murine model.^[^ ^127^ ^]^ Over‐expressed MMP‐2 in tumor tissues plays a significant role in the invasion and metastasis.^[^ ^128^ ^]^ Wang et al. designed a self‐assembly and MMP‐2 responsive nanoplatform to co‐deliver a photosensitizer, IR780, and an anti‐PD‐L1 peptide, APP. Hydrophobic IR780 and hydrophilic APP were connected to the MMP‐2 responsive nanoplatform via a methionine fragment and a PLGLAG peptide, respectively (**Figure** **4**a,b).^[^ ^129^ ^]^ The MMP‐2 responsive delivery system had two features: the nanocarriers were responsive to over‐expressed MMP‐2 in the tumor microenvironment to release the therapeutic agents in tumor cells to minimize their systemic side effect; MMP‐2 responsive cleavage shrank the nanostructures from a size of 151.1–34.12 nm to enhance their deep penetration within the tumor tissues (Figure 4c). ROS generated from IR780 under irradiation of a light induced ICD, exposure of the CRT promoted the maturation of DCs, and the released APP revitalized the effector T cells (Figure 4d) and down‐regulated Tregs (Figure 4e) by blocking the PD‐1/PD‐L1 pathway to improve the antitumor effect. The synergic PDT and immunotherapy via self‐assembly NPs not only directly enhanced their antitumor effect, but also activated the immune system to eradicate untreated distant tumors and prevented the lung metastasis compared with free IR780 or APP without the nanoplatform (Figure 4f,g).

**Figure 5:**
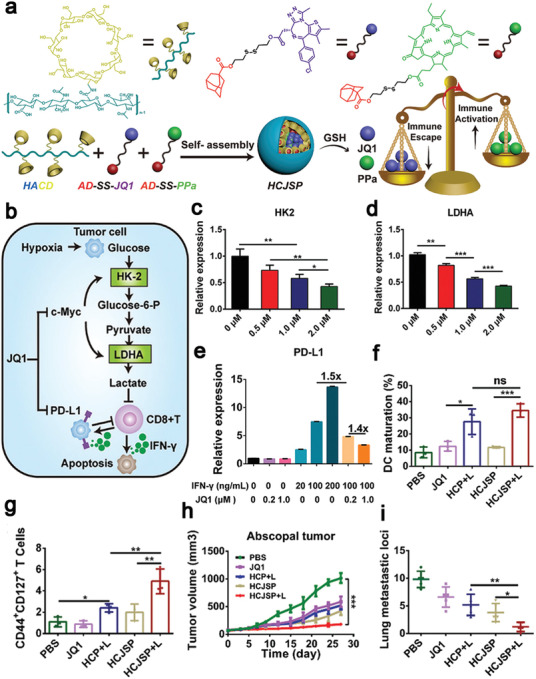
a) Schematic illustration of constructing a supramolecular prodrug nanoplatform from HA‐CD and adamantine‐conjugated heterodimers of JQ1 and Ppa (AD‐SS‐JQ1, AD‐SS‐Ppa) via the host–guest interaction. Schematic diagram of b) the glucose metabolism in tumor cells, JQ1 down‐regulated the expression of c) HK2, d) LDHA, and e) PD‐L1. The synergic therapy promoted f) DCs maturation and g) increased the population of effective memory T lymphocytes, demonstrating effective inhibition of h) abscopal tumor growth and i) lung metastasis. Reproduced under the terms of the Creative Commons CC‐BY license.^[^ ^136^ ^]^ Copyright 2021, The Authors. Published by Wiley‐VCH.

A tumor hypoxia microenvironment weakens the efficacy of PDT.^[^ ^130^ ^]^ First, hypoxia impedes the generation of ROS that can activate the host immunity system.^[^ ^131^ ^]^ Strategies have been proposed to increase the concentration of oxygen within the microenvironment to strengthen the PDT‐based immune responses. For example, nano‐sized metal‐organic frameworks (nMOFs) constructed from Fe_3_O clusters and a ligand of 5,10,15,20‐tetra(p‐benzoato) porphyrin (TBP), which disintegrated H_2_O_2_ to O_2_ through a Fenton‐like reaction under irradiation to address the issue of hypoxia.^[^ ^132^ ^]^ The results exhibited that the nMOFs alleviated tumor hypoxia by directly generating O_2_ and indirectly down‐regulating HIF‐1*α* protein expression. Extraordinarily, the addition of anti‐PD‐L1 antibodies regressed both primary and abscopal tumors. Reduction in hypoxia levels and combination of PDT with anti‐PD‐L1 antibodies could contribute to remarkable cancer regression through strong immunity responses in colorectal cancer. In addition, hypoxia modifies the metabolic pathways in the tumor microenvironment and tumor cells obtain energy through glycolysis rather than oxidative phosphorylation.^[^ ^133^ ^]^ Glycolysis leads to production of lactate that can activate the G‐protein‐coupled receptor GPR81 to suppress innate and adaptive immune cells (NK cells and cytolytic T cells).^[^ ^134^ ^]^ Blocking the oncogene c‐Myc that is involved in regulation of tumor glycolysis is a promising choice for alleviation of immunosuppression.^[^ ^135^ ^]^ *Sun* et al. constructed a supramolecular prodrug nanoplatform via host‐guest complexation. *β*‐cyclodextrin‐grafted hyaluronic acid (HA‐CD) as a host provided a cavity to encapsulate adamantine‐conjugated pyropheophorbide a (Ppa) and bromodomain‐containing protein 4 inhibitor (JQ1) heterodimers as a guest (**Figure** **5**a). JQ1 could be used for regulating transcription of c‐Myc and PD‐L1 and PDT‐mediated ICD significantly regressed primary and distant tumors (Figure 5b), which resulted in prolonging the survival of Panc02‐pancreatic tumor‐bearing mice.^[^ ^136^ ^]^ HA was used for actively targeting tumors via specific binding to the CD44 receptor on the tumor cell surface and prolonging the blood circulation time of the nanoplatform up to 48 h. The GSH‐responsively released JQ1 down‐regulated key glycolytic enzymes (hexokinase‐II (HK2) and lactate dehydrogenase A (LDHA)) and effectively inhibited PD‐L1 expression (Figure 5c–e), which considerably relieved the immune resistance. The combinatory therapy resulted in four and two folds of matured DCs and effective memory T lymphocytes, respectively, in comparison with PBS‐treated group (Figure 5f,g), indicating effective memory immune responses for prevention of abscopal tumor growth and lung metastasis in pancreatic cancer (Figure 5h,i).^[^ ^136^ ^]^ This demonstrated that modification of the tumor hypoxia microenvironment could play a vital role in tumor immunotherapy.

#### 3.2.2 PDT in Combination with IDO Inhibitors

IDO is an enzyme constitutively expressed in human tumors and infiltrating myeloid cells,^[^ ^137^ ^]^ and it can be produced by activated immune cells through stimulation by type I and type II IFN. IDO catalyzes degradation of tryptophan to kynurenine, which could impair survival and activity of CD8^+^ T cells and activate Tregs, leading to suppression of the antitumor immunity.^[^ ^138^ ^]^ Therefore, IDO is a significant mediator, similar to PD‐1/PD‐L1 and CTLA‐4, contributing to reducing peripheral immune responses. IDO inhibitors, such as NLG919 and indoximod, have been pursued for combinational therapies to modulate the immunosuppressive microenvironment.^[^ ^139^ ^]^

**Figure 6:**
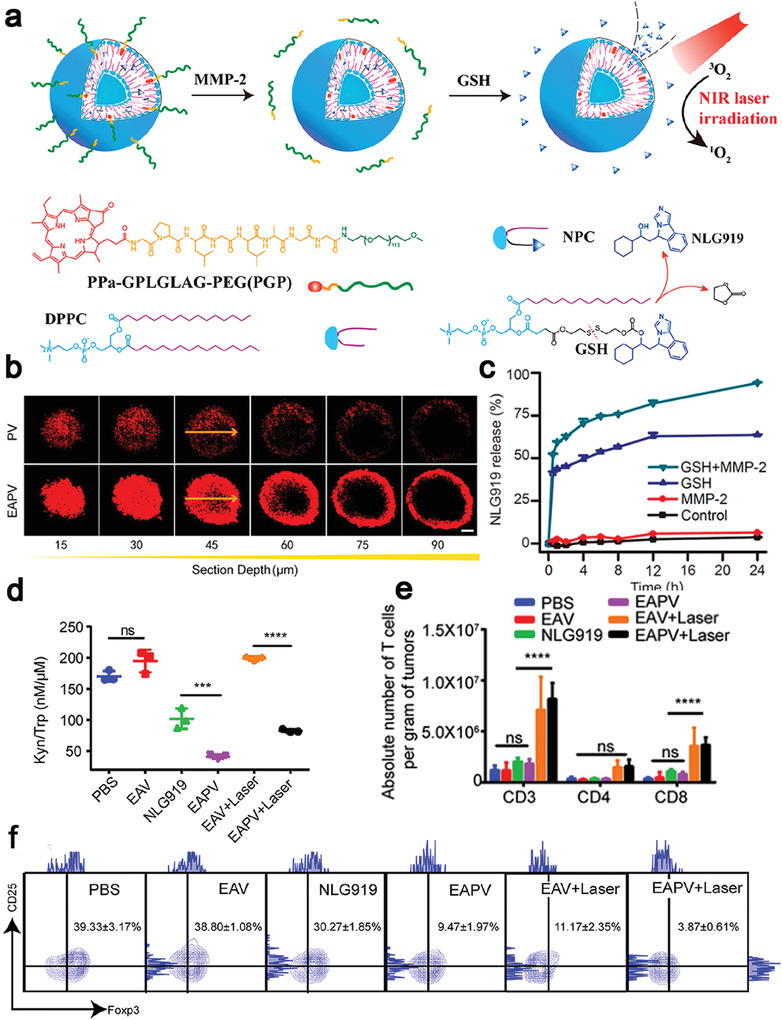
a) Schematic illustration of enzyme activatable prodrug vesicles (EAPVs) for PDT combined with an IDO inhibitor (NLG919). b) Confocal images analysis for revealing the penetration depth of EAPVs on CT26 multicellular spheroids. c) NLG919 release profiles after incubation with 10 mm GSH and 2.5 µg mL^−1^ MMP‐2. d) The ratio of kynurenine to tryptophan on CT26 after different treatments, indicating NLG 919 effectively inhibited the activity of IDO. EAPVs induced ICD, leading to e) an increase in the amount of CD8^+^ T cells and f) a decrease in the population of Tregs in tumor sites. Reproduced with permission.^[^ ^140^ ^]^ Copyright 2019, American Chemical Society.

Gao et al. proposed an MMP‐2/GSH dual‐responsive prodrug to co‐deliver a photosensitizer (Ppa) to induce ICD and an IDO inhibitor (NLG919) to remodel the tumor microenvironment (**Figure** **6**a). Ppa was linked with methoxy poly(ethylene glycol) by a MMP‐2 responsive GALGLPG peptide, and NLG919 was conjugated with palmitoyl lyso‐phosphocholine via a disulfide bond. The prodrug effectively increased the Ppa penetration depth to 45 µm in spheroids (Figure 6b) and notably prolonged the blood circulation time of NLG919.^[^ ^140^ ^]^ NLG919 was released in a GSH‐responsive manner (Figure 6c) to remarkably inhibit degradation of tryptophan to kynurenine (Figure 6d). After intravenous injection, the prodrug was able to increase intratumoral lymphocytes infiltration sevenfold and reduce the Tregs ratio tenfold compared with the PBS‐treated group (Figure 6e,f), dramatically suppressing tumor recurrence especially in the CT26 subcutaneous tumor‐bearing mice.^[^ ^140^ ^]^ To maximize the ICD effect, a pH‐responsive block copolymer of polyethylene glycol‐b‐cationic polypeptide (PEG‐b‐cPPT) was constructed by Yang et al. This copolymer not only formed a self‐assembled nanovesicle, but also served as an ICD inducer. A photosensitizer, 2‐(1‐hexyloxyethyl)‐2‐devinyl pyropheophorbide‐a (HPPH), and an IDO inhibitor, indoximod (IND), were encapsulated within the engineered nanovesicle via hydrophobic interactions. Endosomal escape was facilitated by the positively charged tertiary amines in this nanovesicle to release HPPH and IND into the cytoplasm.^[^ ^141^ ^]^ This nanoparticle delivery system was demonstrated with a great antitumor effect, which could be attributed to three factors: first, HPPH‐mediated PDT generated ROS for directly killing tumor cells and inducing ICD; next, the released IND from the nanovesicle triggered phosphorylation of P‐S6K to activate the mTOR pathway to reverse the tumor microenvironment, which eventually up‐regulated the ratio of stimulated CD8^+^ T cells and restrained Tregs; finally, the nanovesicle not only served as a vehicle to deliver HPPH and IND, but also intrinsically induced CRT translocation from the ER membrane to the cell membrane surface, which led to recruiting DCs into the tumor tissues and provided additional immune stimuli to activate immune responses. The adjuvant effect of the nanovesicle may be due to its endosomal escape ability to activate the NLRP3 inflammasome pathway.^[^ ^142^ ^]^ These contributing factors synergistically resulted in significant apoptosis (55.7%) of B16F10 cells and mitigated the immunosuppressive effects by restoring CD8^+^ T cells.^[^ ^141^ ^]^

### 3.3 ICD by Photothermal Therapy

PTT kills tumor cells by generating hyperthermia in tumor tissues via a photothermal conversion agent under light irradiation in a minimally invasive way. The tumor cells can be ablated at a temperature of 41–48 °C through damaging DNA, denaturing proteins and disrupting the cellular membrane,^[^ ^143^ ^]^ which result in release of tumor‐associated antigens and HSPs as “danger signals”.^[^ ^144^ ^]^ APCs recognize HSPs and present HSPs‐chaperoned peptides to CD8^+^ T cells, which activate the adaptive immunity.^[^ ^145^ ^]^ However, PTT alone cannot be effective in ablating distal and metastasizing tumors due to a short tissue penetration depth of the laser source^[^ ^146^ ^]^ and suboptimal immune activation. Currently, the combination of photothermal conversion agents with immunotherapeutic agents, such as anti‐CTLA ‐4 antibodies,^[^ ^147^ ^]^ anti‐PD‐1/PD‐L1 antibodies,^[^ ^148^ ^]^ IDO inhibitors, and immune adjuvants^[^ ^149^ ^]^ can substantially enhance immune stimulation and relieve the immunosuppressive tumor microenvironment (Table 2).

**Figure 7:**
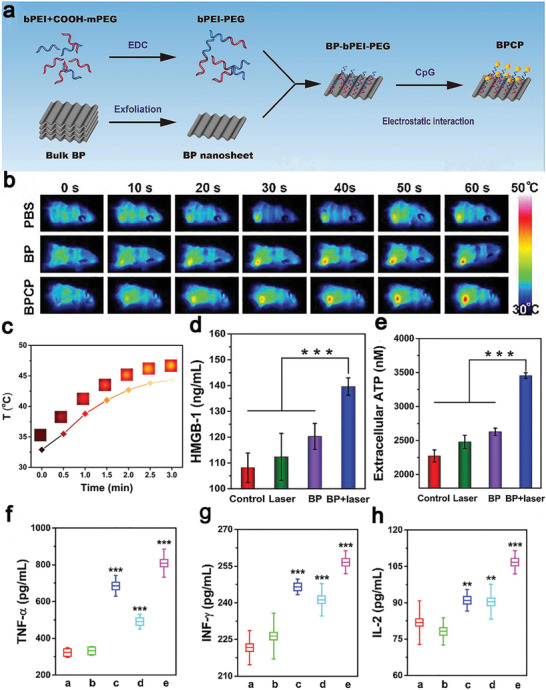
a) Preparation of BP‐based nanocomposites (BPCP) from BP nanosheets incubated with bPEI‐PEG and CpG. b) Infrared thermal images of 4T1 subcutaneous tumor mice after treatment with PBS, BP, and BPCP. c) Temperature rise curves of 4T1 cells incubated with BP nanosheets after NIR laser irradiation. BPCP‐based PTT induced ICD, evidenced with secretion of d) extracellular HMGB1 and e) ATP via ELISA. Serum cytokines levels in mice after different treatments: f) TNF‐*α*, g) IFN‐*γ*, and h) IL‐2 (a: PBS; b: laser; c: CpG; d: BP + laser; e: BPCP + laser). Reproduced with permission.^[^ ^149^ ^]^ Copyright 2020, Elsevier.

A large number of nanostructures including inorganic non‐metallic materials and metallic nanomaterials have strong absorbance in the transparent tissue window under irradiation by an NIR laser and they have been widely applied in PTT. Inorganic nanomaterials such as carbon nanotubes and black phosphorus have shown excellent performance in PTT to activate the immune system. Combination single‐walled carbon nanotubes with anti‐CTLA‐4 antibodies demonstrated its great potential in regression of primary 4T1 breast tumors and their lung metastasis.^[^ ^147^ ^]^ The single‐walled carbon nanotubes were modified with PEG to improve the stability in a physiological solution. The carbon nanotubes had strong absorbance upon irradiation by an 880 nm NIR laser and the temperature of the tumor site was raised up to 53 °C. In addition, single‐walled carbon nanotubes served as an adjuvant to provoke DCs maturation and cytokines secretion both in vitro and in vivo. In addition, Zhao et al. proposed a facile strategy to use an emerging two‐dimensional nanomaterial‐black phosphorus (BP) as an NIR‐responsive nanoconverter for PTT, and an immunogenic adjuvant CpG was grafted on the nanosheets through electrostatic binding to intensify immune responses (**Figure** **7**a).^[^ ^149^ ^]^ The BP‐induced hyperthermia at nearly 45.6 °C (Figure 7b,c) medicated necroptosis to trigger ICD, which translocated the CRT onto the cell membrane surface, released HMGB1 and secreted ATP (Figure 7d,e), thus promoting antigens presentation and secretion of cytokines such as TNF‐*α*, INF‐*γ*, and IL‐2 (Figure 7f–h). As result, BP‐based nanocomposites treatment led to a significantly low tumor growth rate and elimination of discrete tumorlets systemic immune activation induced by PTT and CpG immunomodulation. BP could be an excellent photothermal conversion agent for PTT with a conversion efficiency of 28.41% to raise the tumor temperature to 45 °C as well as great biodegradability and biocompatibility.^[^ ^149^ ^]^

**Figure 8:**
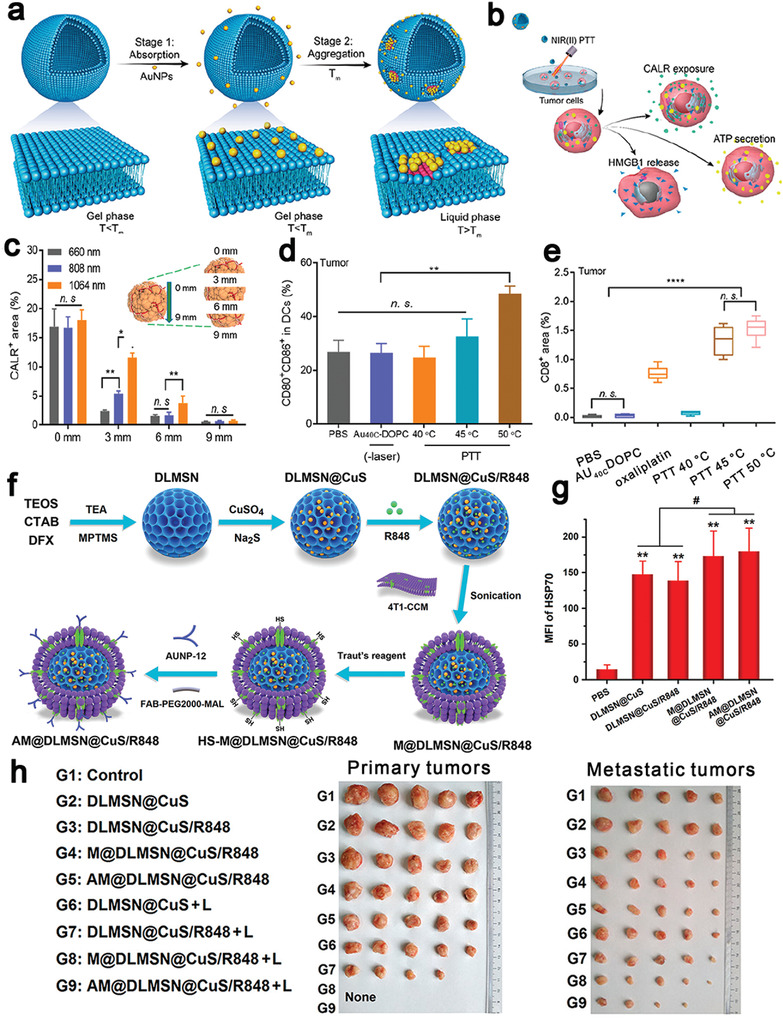
a) Controllable Au NPs aggregation on fluidic liposomes to mediate NIR‐II responsive PTT. b) After irradiation, tumor cells released DAMPs including CRT, HMGB1, and ATP. c) The CRT at different depths of the tumor (0, 3, 6, 9 mm) to demonstrate the great penetration ability of NIR‐II PTT. PTT‐induced ICD to elicit the innate and adaptive immune response, which promoted d) DCs maturation and e) increase the number of CD8^+^ T cells. Reproduced with permission.^[^ ^151^ ^]^ Copyright 2019, American Chemical Society. f) Schematic illustration of the preparation process of the AM@DLMSN@CuS/R848 nanohybrid. Cetyltrimethylammonium bromide (CTAB) and deferred acid (DFX) co‐assembled in an aqueous solution and hydrolyzed by tetraethyl orthosilicate (TEOS) to obtain DLMSNs. g) Confocal fluorescence semi‐quantitative results of HSP 70 in different groups. h) Photographs of primary and metastatic tumors after different treatments two times. Reproduced with permission.^[^ ^155^ ^]^ Copyright 2020, American Chemical Society.

Metallic nanomaterials including gold (Au nanorods, Au nanocages), cuprum and Prussian blue (PB) NPs have also been widely applied as NIR‐absorbing photothermal conversion agents for PTT.^[^ ^150^ ^]^ For instance, Ma et al. demonstrated the great potential of using self‐assembled Au NPs on fluidic liposomes in combination with anti‐PD‐1 antibodies to regress 4T1 tumors (**Figure** **8**a). Photothermal treatment via Au NPs in the second NIR biowindow (NIR‐II) triggered more homogeneous ICD in deeper tumor tissues compared to the first NIR biowindow (NIR‐I) (Figure 8b,c).^[^ ^151^ ^]^ They systematically investigated the underlying immune mechanism: various DAMPs like ATP, CRT, and HMGB1 were secreted from cancer cells after NIR‐II laser irradiation; NIR‐II PTT‐induced ICD facilitated the maturation of DCs and proliferation of CD8^+^ T cells (Figure 8d,e). Efficient control of tumor growth and prevention of tumor metastasis were achieved by stimulating the host immune system. It is noteworthy that the delivery system prominently enhanced CD8^+^ T cells infiltration within tumors up to a depth of 6 mm below the tumor surface, which could address various immune escape issues owing to lack of lymphocytic infiltration in the inner and deep beds of solid tumors by PTT. NIR‐II‐based PTT in combination with imiquimod and anti‐PD‐L1 antibodies effectively regressed lung metastasis of 4T1 tumors. Li et al. constructed a nanohybrid to combine cuprum‐based PTT with an HSP inhibitor to mediate PTT‐based immunotherapy for 4T1 tumor treatment. FeOOH nanodots as an ROS inducer were anchored on Cu‐containing double‐layered hydroxide as a photothermal conversion agent. Cu‐containing double‐layered hydroxide converted the energy from an NIR laser to heat and a fever‐type temperature (40–42 °C) was elevated, leading to CRT exposure on tumor cells. The hyperthermia damage was amplified by ROS production from the Fenton reaction.^[^ ^152^ ^]^ PB NPs could be used as multifunctional vesicles: a photothermal conversion agent for PTT, a nanoenzyme to decompose H_2_O_2_ to O_2_ to relieve hypoxia and a magnetic resonance imaging contrast agent.^[^ ^153^ ^]^ PB NPs were used as a nanostructure for targeted delivery of sorafenib by conjugating PB NPs with a SP94‐targeted peptide for treating hepatocellular carcinoma. Under 808 nm laser irradiation, the temperature rose from 36.2 to 49.2 °C in the HepG2 bearing‐tumor tissue to induce ICD, resulting in releasing tumor associated antigens, CRT, ATP, and HMGB1. PB NPs relieved the hypoxic tumor microenvironment, which decreased the expression of HIF‐*α* and the number of M2 macrophages, while enhanced DCs maturation and CTLs infiltration. PB NPs combined with anti‐PD‐L1 antibodies not only showed prominent antitumor effects in both primary and abscopal tumors, but also induced long‐period immunological memory to inhibit cancer recurrence.^[^ ^154^ ^]^

Since dendritic large‐pore mesoporous silica nanoparticles (DLMSNs) can effectively load cargoes in their large‐sized pores and copper sulfide (CuS) NPs have a high photothermal conversion efficiency for PTT, Cheng et al. constructed a nanoplatform from CuS NPs and DLMSNs. CuS NPs and an adjuvant, resiquimod (R848), were loaded in the pores and inner cavities of DLMSNs for cancer therapy (Figure 8f).^[^ ^155^ ^]^ In addition, a coating layer of cancer cell membranes protected the contents inside DLMSNs and facilitated tumor cells targeting. Furthermore, the protein on the membrane was modified with AUNP‐12, a peptide for inhibiting the PD‐1/PD‐L1 pathway, via thiol‐alkene click reaction. Upon irradiation by a 980 nm laser, the biomimetic and multi‐cargo‐loaded intelligent NPs raised the temperature in the tumor to nearly 60 °C, which remarkably increased the fluidity of the cell membrane to intelligently release R848. The benzoic‐imine bond was ruptured in response to the acidity in tumor cells to release AUNP‐12. After intravenous injection, chain reaction was induced in tumors sites: CuS NPs‐mediated PTT induced ICD to generate antigens and DAMPs (e.g., HSP 70) (Figure 8g) that activated the downstream immune response; R848 and AUNP‐12 remodeled the tumor immune microenvironment to promote DCs maturation, increased the ratio of CD3^+^CD8^+^ CTLs tenfold and enhanced secretion of cytokines like TNF‐*α* and IFN‐*γ*. The nanoformulation treatment led to an increased survival of mice to 47 days and effectively inhibiting the metastasis.^[^ ^155^ ^]^ The nanohybrid‐based approach eradicated a primary tumor and inhibited the abscopal tumor growth (Figure 8h), which provides a new insight into the combinational treatment with nanostructure‐based PTT and immunotherapy.

**Figure 9:**
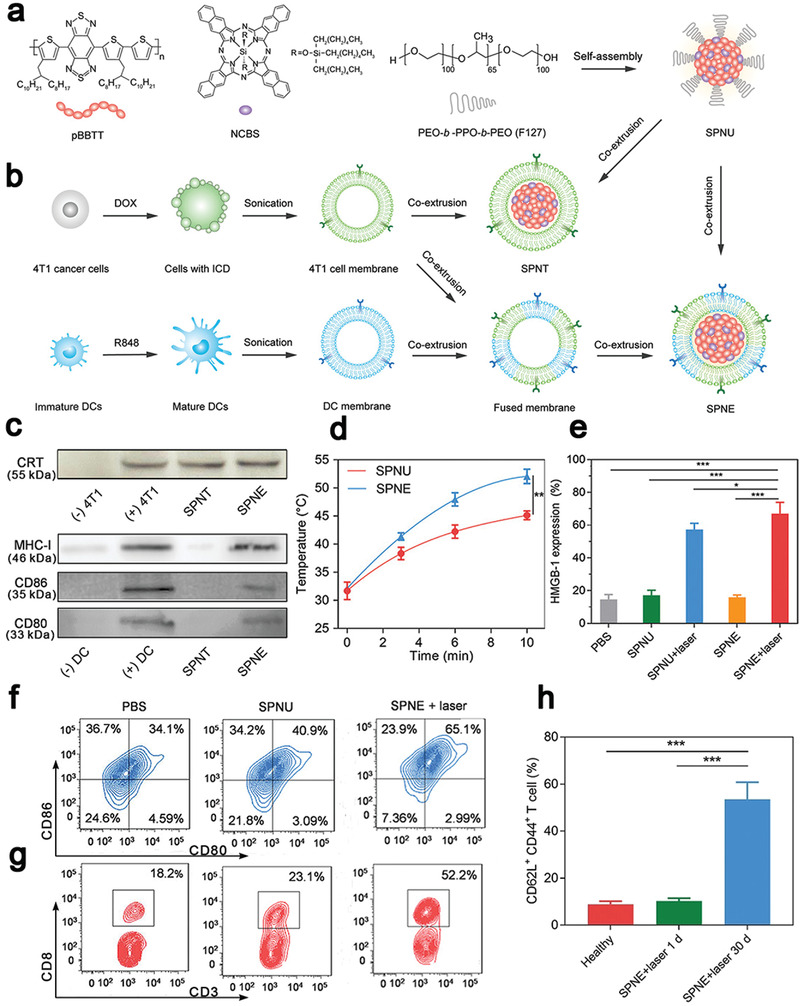
a,b) Schematic illustration of SPNs coated with membranes from 4T1 cells and DCs (SPNE) for PTT‐mediated immunotherapy. c) 4T1 cells were treated with DOX to expose CRT on the membrane surface and DCs were stimulated with resiquimod to provide T cell binding moieties (MHCI, CD80/86). d) The tumor temperature changes under 1064 nm laser irradiation after 24 h administration of SPNE. SPNU were controls without coating cell membranes. e) PTT up‐regulated the expression of HMGB1 in the tumor cytoplasm to induce ICD. f,g) Flow cytometric plots of mature DCs and cytotoxic T cells in lymph nodes after PTT. h) SPNE activated central memory T cells for the long‐term antitumor immunity. Reproduced with permission.^[^ ^158^ ^]^ Copyright 2021, Wiley‐VCH.

Semiconducting polymeric nanomaterials with a high photothermal conversion efficiency in the NIR‐II,^[^ ^156^ ^]^ great biocompatibility, and a size‐independent optical property have been widely applied in PTT.^[^ ^157^ ^]^ Xu et al. constructed semiconducting polymeric nanoparticles (SPNs) from poly(benzobisthiadiazole‐alt‐thiophene) (pBBTT), silicon 2,3‐naphthalocyaninebis(trihexylsilyloxide) (NCBS), and poly(ethylene glycol)‐block‐poly(propylene glycol)‐block‐poly(ethylene glycol) (PEG‐b‐PPG‐b‐PEG) (**Figure** **9**a).^[^ ^158^ ^]^ 4T1 cells were treated with DOX to tag their membranes with DAMPs (CRT) and DCs were stimulated with resiquimod to label their membranes with T cell stimulating factors (major histocompatibility complex I (MHC I) and CD80/86) (Figure 9b,c). Both membranes were coated onto SPNs to form modified SPNs (SPNE), while the uncoated NPs were used as controls (SPNU). After intravenous injection of SPNE, the photothermal conversion efficiency was 88.8% at 1064 nm. SPNE effectively accumulated in both tumors and lymph nodes. The tumor temperature was raised to 52.1 °C after laser irradiation (Figure 9d), which induced a high level of expression of HMGB1 (Figure 9e) to ablate primary and distant tumors in an ICD model. The dual‐cell membrane‐coated SPNE served as a nanovaccine, which increased the mature DCs ratio from 34.1% to 65.1% (Figure 9f) and the population of activated CD8^+^ T cells 2.87‐folds in lymph nodes, in comparison with those in the PBS group (Figure 9g). The treatment induced strong memory immune responses that the population of CD45^+^CD3^+^CD62L^+^CD44^+^ T cells was five times higher than other groups (Figure 9h), effectively preventing tumor recurrence within 30 days. This study provided a new approach for NDDS‐mediated ICD: both ex vivo and in vivo ICD could be combined in a smart NDDS for eliciting strong host immune responses. Similarly, Li et al. confirmed that SPNs were an excellent photothermal conversion agent in the NIR‐II. The temperature of nanoparticle‐containing tumor cells was raised to 51 °C after laser irradiation to induce intense ICD, resulting in releasing tumor‐associated antigens and DAMPs. PTT in combination with a TLR agonist resiquimod that was thermo‐responsively released, led to intense adaptive immune responses to inhibit tumor growth and suppress lung metastasis.^[^ ^159^ ^]^

### 3.4 ICD by Radiotherapy

RT is a mainstream external treatment modality for cancer, and over 50% of cancer patients receive radiation at some point of the treatment.^[^ ^165^ ^]^ RT primarily disrupts the chemical bonds between the bases in DNA and/or intracellular components to kill tumor cells through mitotic catastrophe.^[^ ^166^ ^]^ It has been reported that CRT, HMGB1, and HSP 70 exposed on dead cell surfaces migrate to an extracellular site during RT to elicit ICD.^[^ ^167^ ^]^ Furthermore, RT can induce secretion of pro‐inflammatory cytokines and chemokines, such as type I IFN, IL‐1*β*, TNF, and CXCL16 to stimulate DCs maturation. Both immune‐stimulatory events assist in systemic antitumor immunity in the host to cause the abscopal effect. RT has made considerable advances in tumor treatment, while the immunosuppressive tumor microenvironment can dampen the abscopal effect as RT may up‐regulate the expression of immunity‐inhibiting proteins, such as PD‐L1.^[^ ^168^ ^]^ Therefore, combined RT and immunotherapy may reduce immunosuppression to amplify the antitumor effect (**Table** **3**).^[^ ^169^ ^]^

**Table 3:** ICD induced by radiotherapy and multi‐combinational therapy

ICD inducer	Immunotherapeutic agents	Advantages	Significant outcomes	Model	Ref.
Based on RT					
nMOFs‐enabled radiotherapy	IDO inhibitor (INCB024360)	An extremely low dose of X‐rays to cause efficient tumor regression in a multi‐cancer mouse model	Effective regression of both treated primary tumors and untreated distant tumors in TUBO and CT26 mouse models	CT26 and TUBO breast cancer (Balb/c mice)	^[^ ^171^ ^]^
nMOFs as radiosensitizers	Imiquimod + anti‐CD47 antibodies	Co‐delivery of therapeutic and immunotherapeutic agents, repolarizing immunosuppressive M2 macrophages to immunostimulatory M1 macrophages	Complete eradication of both primary and distant tumors in a bilateral colorectal tumor model	CT26 (Balb/c mice)	^[^ ^172^ ^]^
Hf_6_‐DBA and Hf_12_‐DBA aniline as a radioenhancer	Anti‐PD‐L1 antibodies	Hf_12_‐DBA as an excellent radioenhancer to effectively absorb X‐ray due to a large specific surface area	Effective generation of tumor specific T cells responses to inhibit irradiated tumors and shrink distant non‐irradiated tumors	CT26 (Balb/c mice)	^[^ ^173^ ^]^
^131^I radioisotope	Immunostimulatory CpG and anti‐CTLA‐4 antibodies	Catalase in a hydrogel rapidly formed from a soluble polysaccharide in the presence of endogenous Ca^2+^ to relieve hypoxia	A remarkable synergistic effect to eliminate distant metastatic tumors and long‐term immune memory protection for treated mice	4T1 and CT26 (Balb/c mice)	^[17^ ^6a^ ^]^
X‐ray radiation	Imiquimod, catalase and anti‐CTLA‐4 antibodies	Catalase‐triggered relief of tumor hypoxia and modulation of the immunosuppressive tumor microenvironment	Effective inhibition of the growth of distant metastatic tumors with a very strong abscopal effect and a robust long‐term immune memory effect to protect mice from re‐challenged cancer cells	4T1 and CT26 (Balb/c mice)	^[17^ ^6b^ ^]^
Cu‐based nanoscale coordination polymers (Cu‐NCPs)	Anti‐PD‐L1 antibodies	Cu‐NCPs decomposed H_2_O_2_ to •OH and converted GSH to glutathione disulfide (GSSG)	About 25% of mice with tumor‐free survival and 62.5% distant tumors regressed	CT26 (Balb/c mice)	^[^ ^180^ ^]^
Gadolinium, 5′‐guanosine monophosphate and hemin	Anti‐PD‐L1 antibodies	Gd^3+^‐based nanoscale coordination polymers served as an enhancer of radiosensitization to increase ROS generation, and hemin depleted GSH to intensify oxidative stresses	Inhibition of both primary and distant tumors, and potent immune responses for suppression of lung metastases	CT26 (Balb/c mice)	^[^ ^181^ ^]^
Multi‐modality therapy					
OXA and pheophorbide	Anti‐CD47 antibodies	Dual pH and MMP‐2 responsiveness for target tumor and deep penetration	Delayed growth for 77.8% of the primary tumor, complete regression of the growth of abscopal tumors	B16F10(C57BL/6), CT26 and 4T1 (Balb/c mice)	^[^ ^182^ ^]^
Ce6 and docetaxel	Anti‐CTLA‐4 antibody or anti‐CD47 antibodies	Docetaxel was released in a ROS‐dependent manner	Significantly increased the population of CD8^+^ T cells and proinflammatory cytokines (IFN‐*γ*, IL‐6, and TNF‐*α*), and inhibited distant tumor growth with a high efficiency	Human larynx squamous carcinoma cell (ICR mice)	^[^ ^183^ ^]^
Ppa as a photosensitizer and a ROS‐responsive paclitaxel dimer prodrug	A hydrolysis‐resistant D‐peptide antagonist	Size‐reducible biomimetic NPs by coating red blood membrane for long circulation	Inhibition of the growth of 84.2% tumors after four times treatments, excellent anti‐metastasis effect and negligible damage to major organs	4T1 (Balb/c mice)	^[^ ^184^ ^]^
DOX and HPPH for PDT	Cross‐linked polymersomes as an adjuvant	Amine groups as an adjuvant for DCs maturation by quickly escaping from endosomes	A significant abscopal effect for inhibition of distant tumors	MC38(C57BL/6)	^[^ ^185^ ^]^
DOX and Ce6 for PDT	IDO inhibitor (indoximode)	Laser‐responsiveness, changeable size, on‐demand drug release and prolonged circulation retention	Suppression of the growth of primary tumors, prevention of tumor recurrence and metastasis by inducing robust antitumor cellular immunity responses	4T1 (Balb/c mice) and B16F10 (C57BL/6)	^[^ ^186^ ^]^
OXA and pyrolipid for PDT	Anti‐PD‐L1 antibodies	Biodegradable nanoscale coordination polymer for delivery of multiple therapeutic agents	Regression of primary and distant tumors	CT26 and MC38 (C57BL/6)	^[^ ^187^ ^]^
DOX or OXA	Imiquimod and anti‐PD‐L1 antibodies	Local and sustained release of a “cocktail” chemo‐immunotherapeutic agents	Remarkable promotion of CTLs, an efficient abscopal effect and an immune memory effect	CT26 and 4T1 (Balb/c mice), isogenic mouse glioma (C57BL/6)	^[^ ^188^ ^]^
Photosensitizer rose Bengal and indocyanine green as a light absorber	Anti‐CTLA‐4 antibodies	Upconversion nanoparticles as carriers and lipid molecules DSPE‐PEG‐mal as an antigen‐capturing agent	Long‐term survival of 84% of the treated tumor‐bearing mice and tumor‐specific immunity developed in 34% of mice	4T1 (Balb/c mice)	^[^ ^189^ ^]^
DOX and palladium NPs as a photothermal conversion agent	PD‐L1checkpoint blockade antibodies	Specific accumulation at the tumor site, deep penetration into tumor tissues, and activation in response to the intratumoral enzymatic microenvironment	Efficient improvement CTLs infiltration in the tumor site and an excellent tumor treatment effect for both primary and abscopal tumors	CT26 (Balb/c mice)	^[^ ^190^ ^]^

**Figure 10:**
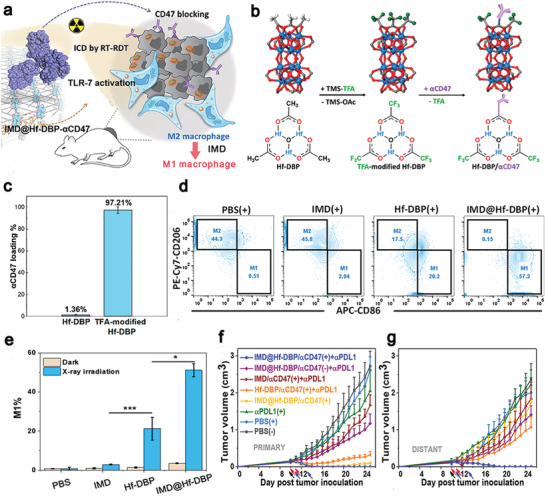
a) Schematic illustration of IMD@Hf‐DBP‐*α*CD47 activated tumor microenvironment to inhibit tumor growth. b) Chemical structures of Hf‐DBP and trimethylsilyl trifluoroacetate (TMS‐TFA)‐modified Hf‐DBP. c) The loading efficiency of anti‐CD47 antibodies in Hf‐DBP and TFA‐modified Hf‐DBP. d,e) Macrophage repolarization ratios after X‐ray irradiation by flow cytometry. f) Primary and g) distant tumor volumes after different treatments. Reproduced with permission from.^[^ ^172^ ^]^ Copyright 2020, American Chemical Society.

nMOFs are excellent radiosensitizers with a high efficiency in loading drugs or macromolecules into porous structures as well as excellent biocompatibility,^[^ ^170^ ^]^ and they have been extensively applied to RT. For example, Lu et al. constructed two hafnium (Hf)‐based nMOFs from 5, 15‐di(p‐benzoato)porphyrin‐Hf (DBP‐Hf) and 5, 10,15,20‐tetra(p‐benzoato)porphyrin‐Hf (TBP‐Hf), and an IDO inhibitor, INCB024360, was encapsulated in the core and channels of nMOFs (termed as IDOi@nMOF). DBP‐Hf acted as an X‐ray absorber for RT for generation of •OH radicals and TBP‐Hf activated the photosensitizers for radiodynamic therapy by generating ^1^O_2_. Both therapies resulted in damaged cell membranes and fragmented double strands of DNA, inducing inflammation and antigen release.^[^ ^171^ ^]^ Treatment with IDOi@nMOF under low‐dose X‐ray irradiation resulted in a significantly higher number of tumor‐infiltrating cytotoxic CD8^+^ T cells and a competent level of inhibition of the catabolism of tryptophan to kynurenine to alleviate T cells exhaustion, which led to an excellent antitumor effect in multi‐tumor models including glioblastoma, head and neck squamous cell carcinoma, prostate and colorectal tumor. The treatment only required an extremely low dose of X‐ray to minimize the side effect of RT, meanwhile enhanced the efficiency of eradicating or regressing both primary tumors in the treatment region and abscopal tumors in the untreated region. Ni et al. used Hf‐DBP nMOFs for RT, in which imiquimod and anti‐CD47 antibodies (termed as IMD@Hf‐DBP/*α*CD47) were co‐delivered to augment the antitumor immunity (**Figure** **10**a,b).^[^ ^172^ ^]^ It was noted that Hf‐DBP treated with trimethylsilyl trifluoroacetate exhibited a remarkable loading capacity of 97.2% for anti‐CD47 antibodies (Figure 10c). The nMOFs‐based RT generated ROS to induce the ICD effect by exposure of CRT on tumor cell surfaces as an “eat‐me” signal, and anti‐CD47 antibodies were released to block the “not‐eat‐me” pathway to promote antigens presentation. Meanwhile, the TLR‐7 agonist, imiquimod, polarized M2 macrophages to pro‐inflammatory (antitumor) M1 macrophages (Figure 10d,e), reversing the immunosuppressive microenvironment. This treatment not only significantly increased tumor‐infiltrating leukocytes, DCs and macrophages, but also increased the ratio of M1 to M2 macrophages, which significantly enhanced the treatment effect to eradicate tumors with a tumor growth inhibition rate of 98.2% and a cure rate of 50%.^[^ ^172^ ^]^ In addition, cooperative treatment of CT26 colorectal tumor‐bearing mice with IMD@Hf‐DBP/*α*CD47 and an anti‐PD‐L1 immune checkpoint inhibitor led to complete ablation of both primary and distant tumors (Figure 10f,g). Furthermore, Hf_12_‐DBA (Hf_12_‐2,5‐di(p‐benzoato)aniline)‐based nMOFs with a large specific surface effectively absorbed X‐ray as an effective radioenhancer. nMOFs incorporated with anti‐PD‐L1 antibodies have been shown encouraging radiotherapeutic effects in CT26 colorectal mice.^[^ ^173^ ^]^ Therefore, nMOFs prepared from heavy‐metal secondary building units and photosensitizing ligands can enhance X‐ray absorption to generate ROS and singlet oxygen (^1^O_2_), which have improved the efficacy of RT and radiodynamic therapy.^[^ ^174^ ^]^ The modified nMOFs could be served as an excellent nanostructure to co‐deliver small molecules and biomacromolecules for combined RT and immunotherapy.

**Figure 11:**
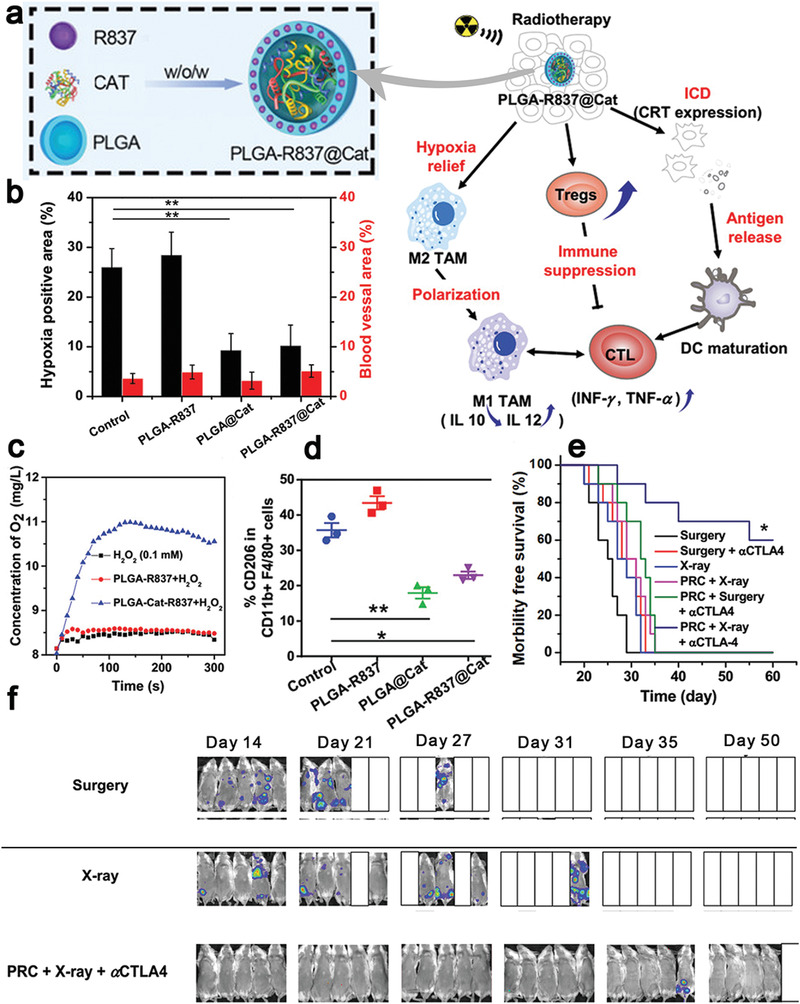
a) Schematic illustration of PLGA NPs for co‐delivery of catalase and imiquimod for RT. Catalase relieved the hypoxia in the tumor microenvironment to intensify the RT effect, and R837, a toll‐like receptor 7 agonist, acted as an adjuvant. b) Hypoxia positive area in tumor slices. c) Oxygen generation by PLGA‐R837 or PLGA‐R837@Cat in a H_2_O_2_ solution (0.1 × 10^−3^ m). d) The percentage of M2 macrophages (CD206^+^) in CD11b^+^F4/80^+^ cells after different treatments. Antitumor effect of PLGA‐R837@Cat based‐RT in combination of an anti‐CTLA‐4 antibody (*α*CTLA4) in 4T1 breast tumors: survival curves of e) the 4T1 tumor metastasis mice; f) spread and growth of fluc‐4T1 cancer cells in different treatment groups. Reproduced with permission.^[^ ^176b^ ^]^ Copyright 2019, Wiley‐VCH.

Rapid proliferation of tumor cells and their abnormal vascular structures often created a hypoxic microenvironment, and the host can repair damaged DNA induced from RT especially at a low concentration of oxygen available in such a tumor microenvironment to diminish the antitumor effect from RT.^[^ ^175^ ^]^ It is a sagacious way to relieve hypoxia by co‐delivery of oxygen suppliers, such as catalase, to decompose over‐produced hydrogen peroxide to oxygen.^[^ ^176^ ^]^ Chen et al. designed multifunctional PLGA NPs to load catalase in its aqueous cavity and imiquimod (R837) in its shell for RT (**Figure** **11**a). Under X‐ray radiation (6 Gy), tumor cells were killed to release cell debris and expose CRT to activate the immunity system. In addition, the loading catalase not only generated oxygen to dramatically alleviate the hypoxia stress (Figure 11b,c), but also evidently reduced the ratio of M2 to M1 macrophages (Figure 11d), reversing the immunosuppressive microenvironment to prolong the survival of 4T1‐bearing mice (Figure 11e). Anti‐CTLA‐4 antibodies were intravenously injected after RT to block CTLA‐4 on T cells, showing a significant effect in prevention of tumor metastases (Figure 11f). This combinational therapeutic approach effectively triggered adaptive immunity responses to regress primary tumors and simultaneously induced immunogenic memory responses to overcome re‐challenge of tumor cells. It can be seen that RT has the ability to activate the immune system, but its synergy with immunotherapy could have notable potential in regression of primary tumors and prevention of abscopal metastases.

**Figure 12:**
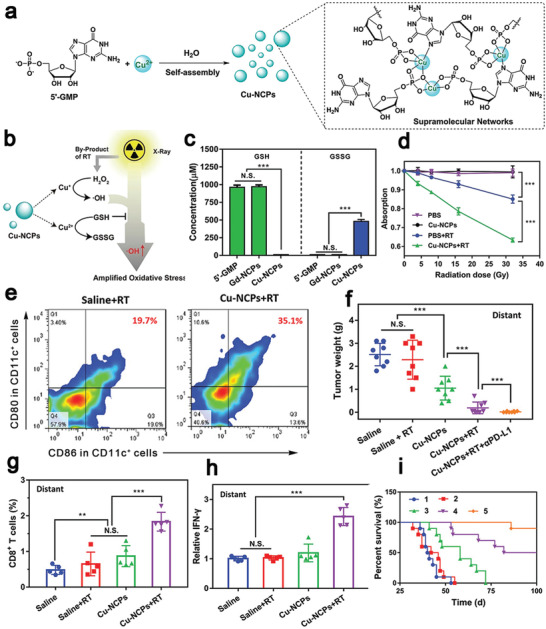
a) Schematic diagram for the assembly process of Cu^2+^ and 5′‐guanosine monophosphate disodium salt to construct Cu‐based nanoscale coordination polymers (Cu‐NCPs), and formation of a supramolecular network from Cu‐NCPs. b) The mechanism of mixed‐valence (Cu^+^ and Cu^2+^) to generate •OH and deplete GSH. (c) Cu‐NCPs converted GSH to GSSG in vitro. d) •OH generation at different radiation doses. e) Representative flow cytometric analysis of DCs maturation in lymph nodes. f) Cu‐NCPs combined with anti‐PD‐L1 antibodies (*α*PD‐L1) effectively inhibited distant tumor growth, increased the population of g) CD8^+^ T cells and h) promoted the secretion of IFN‐*γ* in distant tumors. i) Survival curves of spontaneous triple‐negative breast cancer metastasis‐bearing mice after different treatments. Group 1: Saline, Group 2: Cu‐NCPs + RT + CD8a, Group 3: RT + *α*PD‐L1, Group 4: Cu‐NCPs + RT, and Group 5: Cu‐NCPs + RT + *α*PD‐L1. Reproduced with permission.^[^ ^180^ ^]^ Copyright 2021, Wiley‐VCH.

The efficiency of RT is highly dependent on ROS generation such as hydroxyl radical (•OH),^[^ ^177^ ^]^ while abundant GSH in the tumor microenvironment can rapidly quench •OH to diminish RT‐mediated oxidative stresses^[^ ^178^ ^]^ and weaken the ICD effect.^[^ ^179^ ^]^ A few strategies are proposed to increase the amount of •OH in tumors and deplete GSH in the tumor microenvironment. CuSO_4_ and 5′‐guanosine monophosphate (5′‐GMP) self‐assembled to prepare a mixed valence Cu^+^/Cu^2+^‐based nanoscale coordination polymer (**Figure** **12**a), in which Cu^+^ could decompose H_2_O_2_ to •OH and Cu^2+^ deplete GSH to GSSG (Figure 12b–d).^[^ ^180^ ^]^ This Cu^+^/Cu^2+^‐based RT noticeably intensified the ICD effect, increased the population of matured DCs from 19.7% to 35.1% in lymph nodes (Figure 12e). Combined with anti‐PD‐L1 antibodies, the Cu‐based nanostructure effectively inhibited the growth of distant tumors (Figure 12f) and promoted intratumoral CD8^+^ T cells infiltration and IFN‐*γ* secretion for potent immune responses (Figure 12g,h). The Cu‐based nanostructure displayed an excellent anti‐metastatic effect in a spontaneous triple‐negative breast cancer metastasis model. 50% of mice survived after 100 days, while the survival ratio was elevated to 90% when they received the Cu‐based nanostructure in combination with anti‐PD‐L1 antibodies (Figure 12i). Additionally, Gd^3+^‐based nanoscale coordination polymers could enhance radiosensitization for generation of ROS and the incorporated hemin could deplete GSH to amplify the oxidative stress, resulting in effectively inducing potent immunogenicity at tumor sites.^[^ ^181^ ^]^ Both studies demonstrated that the enhancement in ROS generation and GSH depletion can significantly improve the abscopal effect in RT with effective inhibition of tumor cell proliferation and tumor metastases.

### 3.5 ICD Induced Multi‐Modality Therapy

**Figure 13:**
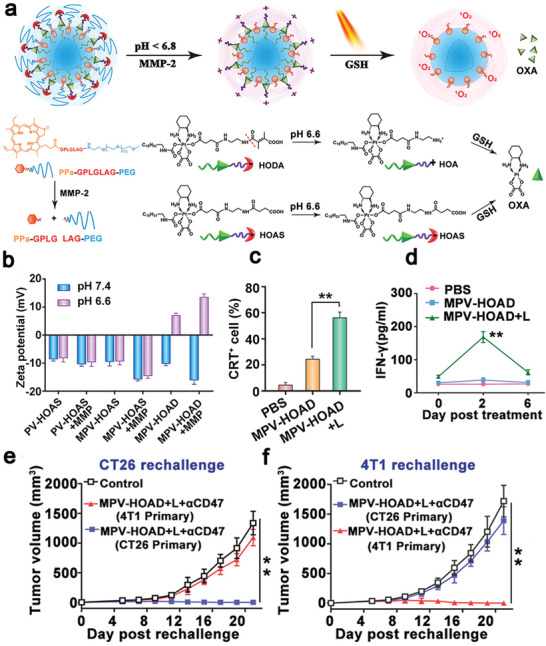
a) Schematic illustration of acidity and MMP‐2 dual‐responsive prodrug vesicles (MPV) for co‐delivery of OXA and a photosensitizer. 2,3‐dimethylmaleic anhydride (DMMA) was used to modify hexadecyl‐oxaliplatin diethylene amine (HOA) to form an OXA prodrug (HOAD) and the photosensitizer was conjugated with PEG via an MMP‐2‐labile GPLGLAG peptide. b) MPV‐HOAD switched from a negative charge to a positive one at pH 6.5 in the presence of MMP‐2. ICD induced by OXA and PDT resulted in c) CRT exposure on the tumor cell surface and d) secretion of IFN‐*γ*. e,f) The multi‐modality therapy activated the immune system of mice for long memory immune responses and protected them from re‐challenge of CT26 and 4T1 live tumor cells. Reproduced with permission.^[^ ^182^ ^]^ Copyright 2019, Wiley‐VCH.

To maximize the antitumor effect, multi‐modality therapeutic approaches have been developed by combining chemotherapy, PDT/PTT, and immunotherapy (Table 3). By rationally designing responsive NDDSs for synchronous delivery of multiple therapeutic agents with different mechanisms, synergistic multi‐modality therapies including chemotherapy and PDT enable reducing the drug dosage to avoid tumor resistance and metastasis with low/no toxicity.^[^ ^186^, ^187^, ^191^ ^]^ Zhou et al. designed an acidity and MMP‐2‐responsive vesicle (MPV‐HOAD) to load an OXA prodrug and a PEGylated photosensitizer (**Figure** **13**a). The surface charge could be switched from negative to positive after striping the PEG corona in responsive to MMP‐2 in the tumor site (Figure 13b), facilitating internalization of vesicles into deep tumor cells.^[^ ^182^ ^]^ Upon laser illumination, both ROS generated from PDT and OXA instantaneously released from the vesicle triggered ICD, which in turn was accompanied with CRT exposure and IFN‐*γ* secretion (Figure 13c,d). Furthermore, anti‐CD47 antibodies for blocking CD47, a “don't eat me” signal, amplified the ICD‐mediated antitumor effect. Chemotherapy with an OXA prodrug, PDT via a PEGylated photosensitizer, and immunotherapy via CD47 blockade not only efficiently regressed primary and distant tumors, but also prevented autologous tumor recurrence in both CT26 and 4T1 tumor models (Figure 13e,f). Wu et al. used amphiphilic diblock copolymers of PEG and polyphosphoester (PPE) with pendant thioether groups to construct an oxidation‐sensitive NDDSs for delivery of Ce6 and docetaxel. After irradiation by a laser at 660 nm, Ce6‐based PDT produced ROS to trigger CRT exposure and docetaxel was released in a ROS‐responsive manner due to a change from hydrophobicity to hydrophilicity in the PPE core.^[^ ^183^ ^]^ Besides these common therapeutic approaches, sonodynamic therapy, gas therapy (e.g., O_2_, NO, and CO), anti‐angiogenesis‐mediated starving therapy or magnetic hyperthermia therapy in combination with immunotherapy have been demonstrated with promising therapeutic effects,^[^ ^192^ ^]^ which have broaden the therapeutic modalities.

**Figure 14:**
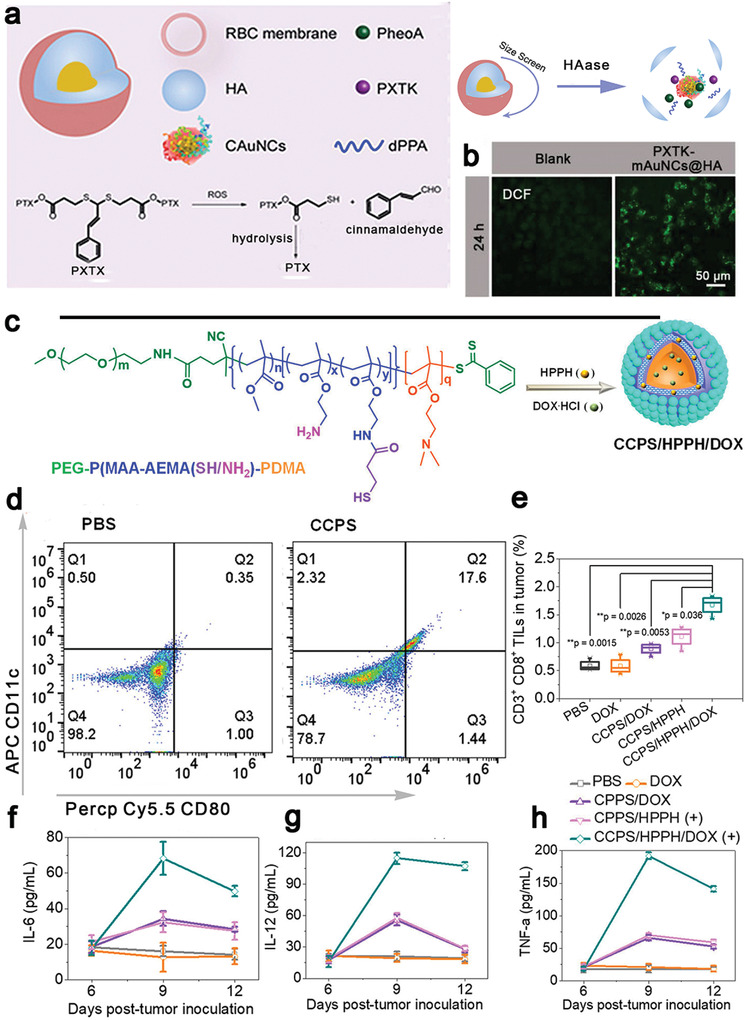
Multi‐modality therapeutic approaches by combining PDT, chemotherapy, and immunotherapy for cancer therapy. a) Illustration of red blood cell membrane‐coated biomimetic size‐reducible NPs, which were degraded into smaller particles in the presence of hyaluronidase. b) ROS generated from cinnamaldehyde stimulated mitochondria to intensify the PDT effect. Reproduced with permission.^[^ ^184^ ^]^ Copyright 2019, Elsevier. c–h) PDT and DOX induced ICD to establish vaccination for CT26 tumor therapy. c) Self‐assembled chimeric cross‐linked polymersomes (CCPS) from polyethylene glycol‐poly (methyl methyacrylateco‐2‐amino ethyl methacrylate (thiol/amine))‐poly 2‐(dimethylamino)ethyl methacrylate (PEG‐P(MMA‐coAEMA (SH/NH2)‐PDMA) served as an all‐in‐one polymersomal nanoformulation with encapsulated HPPH and DOX. d) CCPS acted as an adjuvant to promote DCs maturation. e) CD8^+^ T cells in tumor sites after different treatments. ELISA analysis of serum cytokines f) IL‐6, g) IL‐12, and h) TNF‐*α*. Reproduced with permission.^[^ ^185^ ^]^ Copyright 2019, American Chemical Society.

To promote the antitumor effect, size‐shrinkage of smart NPs could be beneficial for deep penetration to reduce tumor resistance and metastasis. For example, hyaluronic acid (HA), an endogenous polysaccharide with excellent biological safety, is degradable by hyaluronidase in vivo. Yu et al. harnessed this feature to construct size‐reducible NPs that consisted of cationic gold nanoclusters within its core and HA as the outer shell (**Figure** **14**a). To avoid clearance by the mononuclear‐phagocyte system, red blood cell membranes were coated onto NPs for a long circulation time up to 36 h. A chemotherapeutic agent (a cinnamaldehyde and thioacetal‐based PTX dimer prodrug) and a photosensitizer (Ppa) were loaded to the cationic gold nanoclusters. These NPs degraded into smaller particles to effectively penetrate into the tumor tissue up to 90 µm.^[^ ^184^ ^]^ Under irradiation, the ROS‐responsive prodrug released PTX for chemotherapy, and cinnamaldehyde stimulated mitochondria to generate ROS at a high level for enhancing the PDT effect (Figure 14b). Furthermore, a hydrolysis‐resistant D‐peptide antagonist was used to block the PD‐1/PD‐L1 pathway to avoid the immune escape. This tri‐modality therapy resulted in an increase in the number of tumor‐infiltrating CD8^+^ T cells, activation of NK cells, enhancement in secretion of cytokines such as IL‐12 and TNF‐*α*, a tumor inhibition rate of 84.2%, and effective restraint of the lung metastasis.^[^ ^184^ ^]^

This multi‐modality therapeutic approach can also be used to produce a cancer vaccine for personalized treatment. Yang et al. synthesized a copolymer from PEG, poly(methyl methyacrylate‐*co*‐2‐amino ethyl methacrylate (thiol/amine)) (p(MMA‐coAEMA(SH/NH_2_)), and poly 2‐(dimethylamino)ethyl methacrylate (PDMA), which formed triblock copolymeric NPs via a self‐assembly process in an aqueous solution (Figure 14c). DOX and a photosensitizer, HPPH, were loaded into the NPs.^[^ ^185^ ^]^ The polymersomes were multifunctional: first, it had primary, secondary and tertiary amines to facilitate endosome escape and release of pro‐inflammatory factors acting as an adjuvant, which promoted DCs maturation with a 50‐fold higher in the ratio of maturated DCs (Figure 14d) and increased the amount of CTLs at the tumor site (Figure 14e) compared with the PBS‐treated group; DOX and HPPH were released in response to GSH to induce ICD, lead to exposure of CRT and release of HMGB1 by both chemotherapy and PDT; finally, after injection of the polymersomes, the tumor‐bearing C57BL/6 mice secreted a four‐ to ninefold higher level of cytokines (IL‐6, IL‐12, and TNF‐*α* (Figure 14f–h)), and provoked the tumor‐infiltrating lymphocytes population in the tumor, led to inhibiting the growth of both primary and distant CT26 tumors. Furthermore, the DCs vaccination in combination with PD‐1 immune checkpoint inhibitors attenuated T cells exhaustion with a stronger antitumor efficacy.^[^ ^185^ ^]^ Therefore, this tri‐block polymer could be used as an adjuvant as well as a nanoplatform carrier to promote the ICD effect for individualized vaccination for cancer treatment. Functionalized nanocarriers are promising for this multi‐modality therapeutic approach. Huang et al. designed a cyclic seven‐membered ring copolymer with tertiary amines (PCA7) to deliver a model antigen OVA. PC7A activated the STING pathway to sever as an adjuvant, which induced 20‐fold higher CTLs responses compared with free OVA.^[^ ^193^ ^]^ In addition, hydrogel is another effective delivery system for sustained release of therapeutic agents to activate the immune system. A “cocktail” was prepared from a chemotherapeutic agent, DOX or OXA as an ICD inducer, and an immune adjuvant, imiquimod. The cocktail was encapsulated in a hydrogel formed from cross‐linking reaction between alginate and calcium ions.^[^ ^188^ ^]^ Anti‐PD‐L1 antibodies could be directly added into the injection solution to achieve local co‐administration. The “cocktail” therapeutic approach displayed an enhanced antitumor effect for broad tumors, including CT26 colon cancer (OXA as an ICD inducer), 4T1 breast cancer and isogenic mouse glioma (DOX as an ICD inducer). The localized chemo‐immunotherapy achieved an improved therapeutic efficacy for primary and distant tumors and induced effective T cell memory responses to resist re‐challenged pathogens. It is worth mentioning that the preparation process was very simple by mixing these agents to obtain a “cocktail,” which could be suitable for scale‐up production and industrialization, therefore, this “cocktail” approach could have promising potential for clinical translation.

## 4 Future Perspectives

Tumor immunotherapy can activate or boost the immune system to attack tumor cells and elicit specific antitumor immunity and long immunogenic memory responses, therefore, immunotherapy has notably shifted the paradigm of cancer therapy. However, a low response rate from patients due to an immunosuppressive microenvironment is still the main obstacle that hinders broad immunotherapeutic applications. ICD can expose tumor antigens to the APCs and release DAMPs (CRT, HMGB1, ATP, and HSPs) to promote DCs maturation, T cells activation and CTLs infiltration, which result in antitumor immune responses. This ICD‐based therapy in combination with immunotherapy via NDDSs could significantly improve the effect of cancer treatment. This review has summarized recent studies following “CAPIR” principles to engineer NDDSs for induction chemotherapy, PDT/PTT and RT‐based ICD, as discussed in the main text all of these therapy approach elicit potent ICD effect in tumor site which create an immunoresponsive microenvironment to improve the immunotherapeutic effect. These therapeutic approaches have their unique advantages and disadvantages: 1) Chemotherapy is the most common choice for clinic tumor treatment, but it has issues including severe side effects and drug resistance. These issues could be alleviated via NDDSs for target delivery and introducing immunotherapy. 2) PDT or PTT could directly kill tumor cells via cytotoxic ROS or hyperpyrexia in an invasive way, which mostly applied for superficial tumors due to the limited penetration depth.^[^ ^194^ ^]^ Combination with immunotherapy demonstrates effective inhibition of both primary and distant tumors, which may be beneficial for metastatic tumors treatment. 3) RT can trigger antitumor responses far away from the irradiated site via the “abscopal effect” and activate the STING pathway for the innate immunity,^[^ ^195^ ^]^ while these up‐regulated immunosuppressive PD‐1 or M2 macrophages can be inhibited via immunotherapeutic antibodies or polarization agents to maximize therapeutic efficiency.

Encouraged with these promising antitumor effects, a few nanomedicines derived from NDDSs for chemotherapy, PDT/PTT and RT coupled with immunotherapeutic agents (ICIs, cytokines, and immunopotentiators) have entered into clinical trials. Albumin‐bound‐PTX NPs in combination with anti‐PD‐L1 antibody, atezolizumab, could extend the survival period for patients who have metastatic triple‐negative breast cancer. Side effects of this treatment method were found to be well correlated with those in each agent.^[^ ^196^ ^]^ This combinational therapy has been recently approved by FDA as front‐line therapeutic regimen for advanced triple‐negative breast cancer.^[^ ^197^ ^]^ As listed in **Table** **4**, therapeutic treatment with a chemotherapeutic agent, OXA, in synergy with bevacizumab and pembrolizumab is in phase 2 for colorectal cancer metastatic (NCT04262687). OXA is combined with RT and camrelizumab for treating rectal cancer (NCT04558684). Besides, OXA has been combined with nivolumab (anti‐PD‐1) and ipilimumab (anti‐CTLA‐4) for treating advanced non‐small cell lung cancer in phase 2 (NCT04043195). It is particularly worth mentioning that PEGylated Dox‐loading liposomes in synergy with a PD‐L1 checkpoint inhibitor, avelumab, is in phase 3 for treating ovarian cancer with 566 participants (NCT02580058). Currently, clinical trials are focused on combination of ICD‐based therapies with immunotherapy such as anti‐PD‐1/PD‐L1 and anti‐CTLA‐4, which may be the mainstream for tumor therapy in future.

**Table 4:** Clinical trials of ICD‐based therapy synergized with immunotherapy

ClinicalTrials.gov identifier	Therapeutic agents	Immunotherapeutic agents	Tumor	Recruitment status
NCT01637532	Carbo/Caelyx or doxorubicin	Tocilizumab (anti‐IL‐6) and IFN‐*α*	Recurrent ovarian cancer	Completed; phase 2
NCT02406183	Radiation therapy	Ipilimumab (anti‐CTLA‐4)	Melanoma	Completed; phase 1
NCT03380130	Selective internal radiation therapy	Nivolumab (anti‐PD‐1)	Hepatocellular carcinoma	Completed; phase 2
NCT05082259	ASTX660	Pembrolizumab (anti‐PD‐1)	Triple negative breast cancer	Not yet recruiting; phase 1
NCT05034055	Radiation	Atezolizumab (anti‐PD‐L1) and Tiragolumab (anti‐TIGIT)	Non‐small cell lung cancer	Not yet recruiting; phase 2
NCT05019534	Vemurafenib oral tablet	Camrelizumab (anti‐PD‐1) and Cetuximab (anti‐EGFR)	BRAF V600E‐mutated/MSS metastatic colorectal cancer	Recruiting; phase 1
NCT04262687	Capecitabine and Oxaliplatin	Pembrolizumab (anti‐PD‐1) and Bevacizumab (anti‐VEGF)	Colorectal cancer metastatic High immune infiltrate Microsatellite stable	Recruiting; phase 2
NCT04558684	Oxaliplatin, Capecitabine, and radiotherapy	Camrelizumab (anti‐PD‐1)	Rectal cancer	Active, not recruiting; phase1/2
NCT03944252	Cetuximab	Avelumab (anti‐PD‐L1)	Squamous cell anal carcinoma	Unknown; phase 2
NCT05307198	Oxaliplatin and Capecitabine	Sintilimab (anti‐PD‐1)	Rectal neoplasms	Not yet recruiting; phase 2
NCT03388190	5‐Fluorouracil, oxaliplatin and leucovorin	Nivolumab (anti‐PD‐1)	Colorectal cancer metastatic	Active, not recruiting; phase 2
NCT03801304	Vinorelbine	Atezolizumab (anti‐PD‐L1)	Non‐small cell lung cancer	Active, not recruiting; phase 2
NCT03206073	Pexa‐Vec	Tremelimumab (anti‐CTLA‐4) and Durvalumab (anti‐PD‐L1)	Colorectal cancer	Active, not recruiting; phase 2
NCT04043195	Oxaliplatin	Nivolumab (anti‐PD‐1) and Ipilumumab (anti‐CTLA‐4)	Advanced non‐small cell lung cancer	Recruiting, phase1/2
NCT04463368	Melphalan	Nivolumab (anti‐PD‐1) and Ipilumumab (anti‐CTLA‐4)	Uveal melanoma	Recruiting; phase 1
NCT02865811	Pegylated liposomal doxorubicin	Pembrolizumab (anti‐PD‐1)	Ovarian cancer; fallopian tube cancer; peritoneal cancer	Active, not recruiting; phase 2
NCT02580058	Pegylated liposomal doxorubicin	Avelumab (anti‐PD‐L1)	Ovarian cancer	Active, not recruiting; phase 3

Although the NDDSs to induce ICD show promising immunotherapy responses rate, a few challenging issues remain to be addressed. First, therapeutic agents exert their effect on both tumor and immune cells. The balance of activation and inhibition highly depends on the dose and schedule of administration. For example, cyclophosphamide has a different regulating effect on immune cells at a low or high dose and a different treatment schedule.^[^ ^198^ ^]^ Therefore, it is important to choose an optimal dose and metronomic treatment schedules for this combinational treatment approach. The immune responses of RT is tightly correlated with the radiation dose and fractionation regimens,^[^ ^199^ ^]^ and an optimal RT therapy approach could be developed for beneficial immunostimulatory effects but with minimal immunosuppression. Second, cell culture systems and tumor models in most studies are distinctively different from the orthotopic tumor. The tumor microenvironment contains not only tumor cells with different phenotypes, but also stroma cells, immune cells and an extracellular matrix. Hypoxia and a high interstitial fluid pressure are often found in this tumor microenvironment. However, in vitro cell culture systems, tumor cells with a single phenotype were used and abundant oxygen and energy are provided, which may not mimic the tumor microenvironment in the human being. Furthermore, subcutaneous tumor models are often established for in vivo antitumor studies. The tumor microenvironment and tumor metabolism are very different from those in orthotopic tumors. To fill the gap, 3D in vitro oncology models are developed to closely match the human tumor tissues.^[^ ^200^ ^]^ A glioblastoma multiforme 3D model was established for long‐term evaluation of the therapeutic efficacy.^[^ ^201^ ^]^ With multi‐disciplinary development, a humanized mouse model may provide better prediction of therapeutic outcomes of ICD‐based therapies in combination with immunotherapy. Thirdly, the exact mechanisms of ICD need to be unveiled and new NDDSs could be developed based on these mechanisms. For example, ATP is an important immunogenic messenger in ICD, which could be hydrolyze by ectonucleotidases such as CD39 and CD73 to adenosine for activating G protein‐coupled receptors, leading to immunosuppressive effects.^[^ ^202^ ^]^ Accordingly, anti‐CD39 or CD73 antibodies may have excellent performance in suppressing tumor growth via relieving the immunosuppressive environment.^[^ ^203^ ^]^ Further, autophagy plays an important role in secretion of ATP.^[^ ^204^ ^]^ Treatment with an autophagy inducer, STF‐62247, in synergy with OXA markedly inhibited CT26 tumor growth.^[^ ^205^ ^]^ Besides available ICD inducers, new triggers based on the mechanism of ICD could be explored for new NDDSs. For example, calcium carbonate (CaCO_3_) NPs incorporating curcumin were able to overload mitochondrial Ca^2+^ to generate ROS, thus inducing strong ICD for inhibition of tumor growth.^[^ ^206^ ^]^ Hydrogen peroxide generated from oxidation of glucose via glucose oxidase could be converted to ROS for inducing ICD and exerting ER stresses.^[^ ^207^ ^]^ Finally, although many sophisticated and multifunctional NDDSs have been designed to load therapeutic agents for inducing ICD and realizing immunotherapy, quality control, scale‐up production, and shelf‐life of therapeutic products from NDDSs should also be considered for translation of them into clinical treatment. To broaden the benefit of this combinational therapy using NDDSs to more patients, the principle “keeping it simple” is preferred in industry. Simple designs with a great potency for these NDDSs should be pursued to accelerate their clinical translation. With interdisciplinary cooperation to address these issues, NDDSs‐derived ICD inducing therapy in combination with immunotherapy would provide great promise in effective, accurate, and personalized treatment for advanced cancer in clinics.

## Conflict of Interest

The authors declare no conflict of interest.

## Author Contributions

Z.L. and X.L.: Writing the manuscript and designing the figures. S.F., L.R., and H.C.: Conceiving the manuscript. H.Z.: Writing‐review and editing. X.M.: Supervision. Z.G.: Supervision. K.L.: Conceptualization, funding acquisition, writing‐review and editing, supervision.
